# Synthesis and Performance of Biobased Surfactants Prepared by the One-Pot Reductive Amination of l-Arabinose and d-Galacturonic Acid

**DOI:** 10.1021/acssuschemeng.3c03753

**Published:** 2023-11-01

**Authors:** Laura M. Jansen, Kim W. M. van Rijbroek, Pieter C. den Bakker, Dimphna J. Klaassen-Heshof, Wiert J. B. Kolkman, Niek Venbrux, Vienna Migchielsen, Joost Hutzezon, Wouter B. Lenferink, Sebastian Lücker, Adeline Ranoux, Harry W. C. Raaijmakers, Thomas J. Boltje

**Affiliations:** † Department of Synthetic Organic Chemistry, Institute for Molecules and Materials, Radboud University, Nijmegen 6525 AJ, The Netherlands; ‡ Cosun RD&I, Cosun Innovation Center, Dinteloord 4671 VA, The Netherlands; § Department of Microbiology, Radboud Institute for Biological and Environmental Sciences, Radboud University, Nijmegen 6525 AJ, The Netherlands

**Keywords:** arabinose galacturonic acid, sugar beet pulp, biobased, surfactants

## Abstract

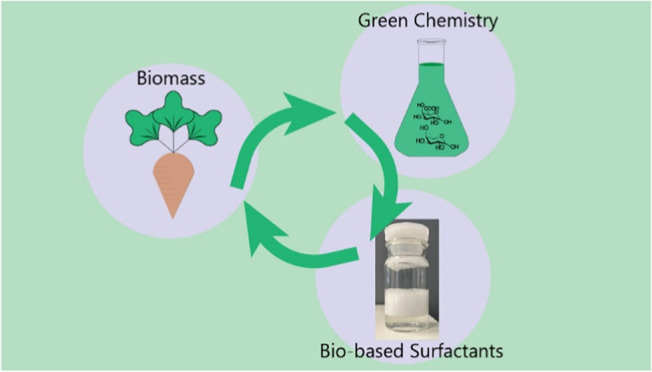

Herein, we report a method for the synthesis of biobased surfactants derived from sugar beet pulp (SBP) monosaccharides, l-Ara and d-GalA. The surfactants were prepared via one-pot reductive amination, allowing the introduction of different alkyl chain lengths and methyl modifications. Optimal reaction conditions were established to achieve high yields and easy purification. The synthesized surfactants including the tertiary amines exhibited desirable properties, including solubility, foamability, and reduction of surface tension. Notably, the anionic surfactants derived from d-GalA demonstrated better solubility and foam performance compared to those derived from l-Ara. In addition, these surfactants exhibited surface tension and critical micelle concentration (CMC) comparable to those of the commercial surfactant sodium lauryl ether sulfate (SLES). Furthermore, the biodegradable surfactant **GalA1.8** displayed excellent emulsifying properties and low skin irritation potential. On the l-Ara surfactant with a short chain, **Ara1.6** has potential as a hydrotrope. These findings suggest that biobased surfactants derived from SBP monosaccharides have promising applications in various industries, including pharmaceuticals, cosmetics, detergents, and chemicals.

## Abstract 2

Herein, we report the synthesis of biobased surfactants derived from the sugar beet pulp-derived monosaccharides l-Ara and d-GalA using a simple and environmentally benign process. The surfactant properties of the prepared derivatives offer versatile potential applications in cosmetics and detergents.

## Introduction

The utilization of biomass has become a subject of great interest as a sustainable alternative for products derived from nonrenewable resources.^1^ Biomass-based products also contribute to the shift toward a more circular economy, which aims to keep all materials and components at their highest utility and value at all times.^2^ Besides a reduced environmental impact, biobased products are potentially also less toxic and more readily biodegradable.^3,4^ In this respect, agricultural byproducts are a useful source of biomass because they cannot be used for food production. Hence, this source of biomass does not interfere with food production and does not require additional arable land.^5^ Agricultural side streams are available at a large scale in the form of residues produced during cultivation, harvesting, and processing of crops. Depending on the feedstock, biomass is available in the form of leftover straw, roots, leaves, cobs, pulps, peels, and seeds.^6^

**Figure 1:**
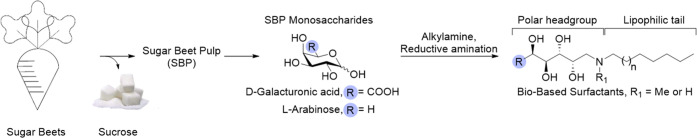
Sugar beet pulp (SBP) monosaccharides and their utilization to prepare biobased surfactants.

Sugar beet pulp (SBP) is an interesting source of agricultural waste, which is obtained during the production of sucrose (Figure 1).^7^ Yearly, sugar beets are being processed into 36.4 million metric tons of sucrose worldwide. During this process, an equal amount of SBP is obtained, which is a cheap and abundant source of biomass.^8,7^ Moreover, SBP is particularly attractive because its utilization does not affect food production to an appreciable extent. Currently, SBP is being sold as animal feed, has a low commercial value, and is rich in polysaccharides such as pectin and cellulose and hemicellulose. These polysaccharides can be enzymatically processed to provide various monosaccharides such as d-galacturonic acid (d-GalA) and l-arabinose (l-Ara).^9,10^ Efforts directed at the large-scale recovery of pure d-GalA and l-Ara may enable their production at large scale in a sustainable manner.^11^ This makes them an interesting starting material for the production of high-value commercial products.^1,12,13^

The hydrophilic nature of monosaccharides enables their application as the polar headgroup for the synthesis of biobased surfactants.^1,14^ Surfactants or surface active agents have the ability to reduce the surface tension and are therefore essential ingredients in the pharmaceutical, cosmetic, detergent, and chemical industries.^14,15^ Microbial biosurfactants such as rhamnolipids and sophorolipids can be produced in various microorganisms and from different feedstocks.^16^ Carbohydrate-based surfactants can also be prepared via chemical synthesis from monosaccharides isolated from biomass. The commercial production of carbohydrate-based surfactants started in the 1980s and is mostly based on sucrose and glucose derivatives.^17−21^ Much less is known about surfactants derived from l-Ara and d-GalA, monosaccharides that can be obtained from SBP in bulk quantities without direct competition with food production.^18,22,23^

Herein, we report a simple, green method to prepare biobased surfactants derived from the SBP monosaccharides d-GalA and l-Ara via one-pot reductive amination. Different chain lengths of alkylamines were introduced using this method, leading to a variety of secondary amine products. Using a second hydrogenation with formaldehyde, novel surfactants containing a tertiary amine were also prepared. In particular, we found that the tertiary amine surfactants are more soluble in water and display favorable surfactant properties such as foamability, stable foams, and emulsions, lowered surface tension of solutions, and a variable critical micelle concentration.^24,25^ Hence, this class of molecules may represent a promising set of biobased surfactants derived from sugar beet pulp.

## Results and Discussion

Reductive amination of carbonyl-containing compounds is an effective method to form C–N bonds.^26,27^ This reaction can be used to selectively modify monosaccharides by making use of the aldehyde present in the open form with secondary or tertiary amines.^28,29^ Several sugar monomers converting into their corresponding amines via reductive amination reactions have been reported.^30^ For example, a reaction with d-glucose, methylamine, and a reducing agent affords *N*-methylglucamine, a precursor in the synthesis of Glucopure.^23,31,32^ In contrast to glucose-based surfactants, much less is known about the reductive amination reactions with the SBP monosaccharides l-Ara and d-GalA.^19^ Hence, we set out to investigate the synthesis and properties of biobased surfactants based on l-Ara and d-GalA formed by the reductive amination with various alkylamines.

We started with exploring the optimal conditions to synthesize biobased surfactants derived from d-GalA via reductive amination using primary or secondary amines (see Pages S2–S8 for experimental details). We will refer to the compounds according to their monosaccharide abbreviations, l-arabinose (Ara) and d-galacturonic acid (GalA), followed by a number denoting the optional presence of a methyl group (1) and the length of the alkyl chain (6–12). Reaction conditions were optimized to develop an efficient and environmentally benign process (Table 1). As a starting point, we used known conditions, d-GalA, two equivalents of octylamine and NaBH_4_ in MeOH (Table 1, entry 1).^19^ A high yield of the reductive amination product **GalA8** (91%) was obtained using precipitation as a purification method. Under the same conditions, a secondary amine was used to afford the corresponding tertiary amine **GalA1.8**. However, this product was obtained in a much lower yield (19%), which was likely the result of its higher solubility and hence poor recovery using the precipitation protocol (Table 1, entry 3). To improve the yield of **GalA1.8** and enable purification by filtration, we employed palladium on carbon and hydrogen gas to carry out reductive amination. Reductive amination of d-GalA monohydrate with *N,N*-methyloctylamine under these conditions led to a much improved yield (88%) of **GalA1.8** (Table 1, entry 4). Even though the monosaccharide and secondary amine were used in 1:1 molar ratio, the reaction occurred till full conversion, and the catalyst could be simply removed by filtration. **GalA8** was also obtained via these conditions (Table 1, entry 2) but in a much lower yield (26%). Since **GalA8** has a much lower solubility than that of **GalA1.8**, the product was likely retained on the filter together with the catalyst during the filtration step. Intrigued by the higher solubility of tertiary amine **GalA1.8** vs the secondary amine **GalA8**, we set out to optimize the synthesis of **GalA1.8** further. Secondary amines such as *N,N*-methyloctylamine are much more expensive and less available than the corresponding primary alkyl amines. Hence, we explored the use of a one-pot two-step synthesis to prepare **GalA1.8** from cheap starting materials, octylamine and formaldehyde (Table 1, entry 5–7). In the first reductive amination step, octylamine was reacted with d-GalA using Pd/C and H_2_ to afford **GalA8**. Upon the complete conversion of d-GalA, formaldehyde was added to afford **GalA1.8** in 97% overall yield (Table 1, entry 5). Overall, the process did occur within a long reaction time of 48–96 h. Hence, to shorten the reaction time, the two-step reaction was run at 35 °C instead of room temperature, leading to 88% within 48 h (Table 1, entry 6). Finally, the solvent was changed to ethanol to make the reaction more environmentally benign because bioethanol can be used as well, to afford **GalA1.8** in 99% overall yield.

**Table 1:**
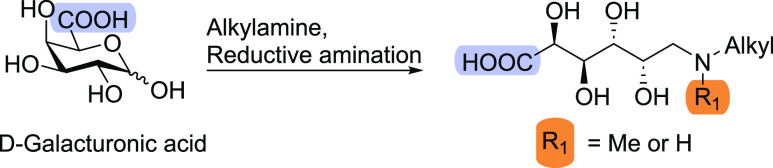
Optimization of Reaction Conditions of 1-(*N-*Methyl-*N*-octyl)-d-galactaric Acid Amine

entry	condition step 1	condition step 2	product	yield %
1	octylamine (2 equiv), NaBH_4_, 0 °C - rt, MeOH	N.A.	**GalA8**: R_1_ = H	91
2	octylamine (1 equiv), Pd/C, H_2_, rt, MeOH	N.A.	**GalA8**: R_1_ = H	26
3	*N,N*-methyloctylamine (2 equiv), NaBH_4_, 0 °C - rt, MeOH	N.A.	**GalA1.8**: R_1_ = Me	19
4	*N,N*-methyloctylamine (1 equiv), Pd/C, H_2_, rt, MeOH	N.A.	**GalA1.8**: R_1_ = Me	88
5	octylamine (1 equiv), Pd/C, H_2_, rt, MeOH	formaldehyde (3.0 equiv)	**GalA1.8**: R_1_ = Me	97
6	octylamine (1 equiv), Pd/C, H_2_, 35 °C, MeOH	formaldehyde (3.0 equiv)	**GalA1.8**: R_1_ = Me	88
7	octylamine (1 equiv), Pd/C, H_2_, 35 °C, EtOH	formaldehyde (3.0 equiv)	**GalA1.8**: R_1_ = Me	99

With the optimal reaction conditions established, we set out to explore the synthesis of a panel of biobased surfactants using l-Ara and d-GalA as starting materials (Table 2). These monosaccharides were used to provide two polar head groups (**Ara** and **GalA**). Moreover, two other modifications were introduced: a variety of alkyl chain lengths and R_1_ may or may not be methylated. Due to the commercially available *N*-methylalkyl chains (R_1_ = Me) with 6, 8, and 12 carbon chains, these were used to react with the two sugars to obtain six different surfactants. Using the reactions conditions of Table 1, entry 4, **Ara1.6 Ara1.8**, and **Ara1.12** were synthesized in 85–95% yield with **GalA1.6, GalA1.8**, and **GalA1.12** synthesized in 86–90%. The *N*-methyldecyl chain was not commercially available; hence, the corresponding surfactants were made via a one-pot two-step synthesis starting from the respective commercially available primary amine (Table 1, entry 7). Under hydrogenation conditions, l-Ara and d-GalA were reacted with decylamine and formaldehyde to afford **Ara1.10** (98%) and **GalA1.10** (73%). To investigate the properties of the methyl modification on the surfactants (R_1_ = Me), previously described reference surfactants were synthesized including an *N-*alkyl chain (R_1_ = H).^22^ Primary alkyl amines with 6, 8, 10, and 12 carbon chain length were used to react with l-Ara and d-GalA. Because poor solubility purification was difficult, some of them were synthesized via the previously described method (Table 1, entry 1). In conclusion, most surfactants without methyl (R_1_ = H) were obtained in lower or similar yields than with methyl modification (R_1_ = Me).

**Table 2:**
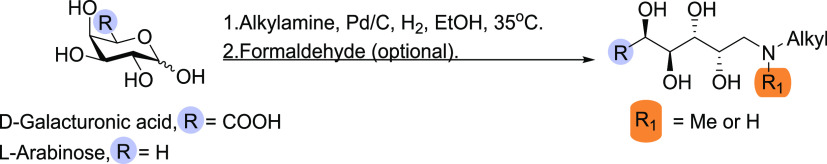

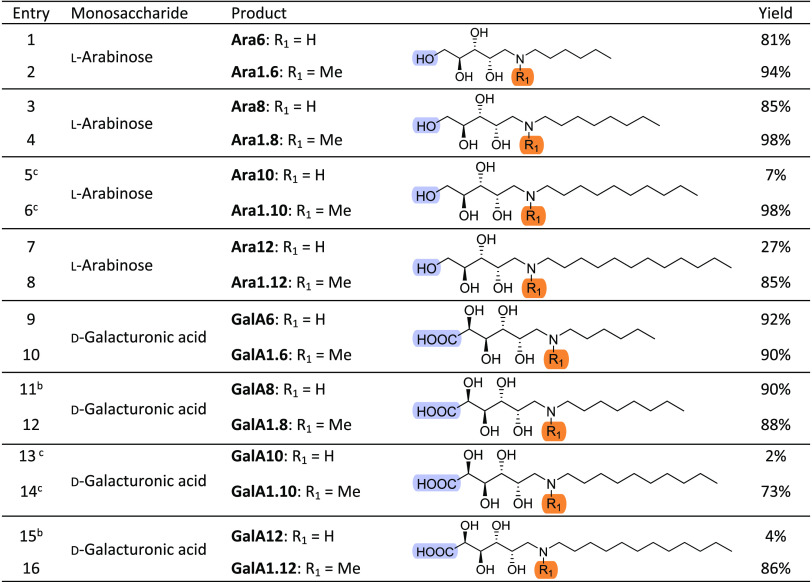
Reductive Hydrogenation of l-Ara and d-GalA to Produce New Biobased Surfactants

a Compounds obtained via reductive amination reactions with conditions of Table 1, entry 4.

b via Table 1 entry 1.

c via Table 1 entry 7.

To establish the optimal application of the prepared surfactants, their properties were established (see Page S9 for experimental details). Various physicochemical properties such as solubility, foamability, surface tension, and critical micelle concentration (CMC) were determined (Table 3).^24^ Since most cleaning formulations are water-based, a high water solubility of the surfactant is desired.^33^ The synthesized surfactants with l-Ara and d-GalA contain an alkyl chain length varying from 6 to 12 carbons. As expected, an increase in the alkyl chain length led to a reduced solubility. In addition, d-GalA-derived surfactants are more soluble compared to their l-Ara counterparts, which can be explained by the anionic and hence more polar nature of the d-GalA residue. An interesting example is a 15 times increase in the solubility of **GalA1.8** compared to that of **Ara1.8**. Moreover, the solubility of the non*-N*-methylated surfactants (R_1_ = H) was determined to compare them with their methylated counterparts (R_1_ = Me). All methylated compounds (R_1_ = Me) showed an increased solubility compared to their corresponding nonmethylated counterparts (R_1_ = H). An interesting example is the solubility of **Ara1.6**, which is 30% and much higher than that of non*-N*-methylated **Ara6** (0.75%). We presume that this effect is caused by the disruption of surfactant stacking by the addition of the methyl group, which in turn increases the solubility of the surfactant.

**Table 3:**
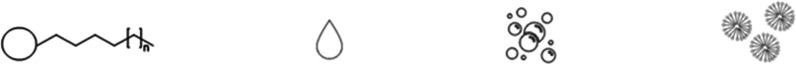
Physicochemical Properties of the Synthesized Surfactants: Solubility, Foamability, Surface Tension, and CMC^a^

				foamability (mL) after (min)				
compound	alkyl chain length	R_1_	solubility (%)	1	5	10	surface tension (mN/m)	CMC (%)
Ara6	6	H	0.75					
Ara1.6	6	Me	30	2	0.2	0	31.4	2
Ara8	8	H	NS					
Ara1.8	8	Me	0.9	5	4.5	4	28.7	0.25
Ara10	10	H	NS					
Ara1.10	10	Me	0.1	3	3	2	33.7	0.02
Ara12	12	H	NS					
Ara1.12	12	Me	0.06	5	5	4	27.8	0.01
GalA6	6	H	14					
GalA1.6	6	Me	40	0.2	0	0	42.1	
GalA8	8	H	1					
GalA1.8	8	Me	15	4.5	4	4	32.1	1.15
GalA10	10	H	0.55					
GalA1.10	10	Me	2	6	6	5	32.85	0.06
GalA12	12	H	0.3					
GalA1.12	12	Me	1	4.5	5	3	33.9	0.1
SLES	12		70	4	4	3	33.8	0.2
Glucopure wet	8–10		4.7	7	5.5	5	27.08	0.03
Glucopure deg	12–14		NS	4	3	3	27.79	0.01

a NS is not soluble (<0.01%)

The foamability and surface tension of the more soluble and interesting methylated surfactants (R_1_ = Me) were determined. Foam was created in a measuring cylinder with an Ultraturrax, and the foam volume (in mL) was monitored over 30 min (Table 3, middle column). The liquid sample had 5 mL starting volume, and the foam volume was measured above the solution in a measuring cylinder. As expected, surfactants with a short alkyl chain did not form a stable foam, such as the six-carbon chain molecules **Ara1.6** and **GalA1.6**. Optimal foaming performance was found for the surfactants with 8, 10, and 12 carbon alkyl chains. All of them generated a foam volume between 3 and 5 mL, which stayed stable over the 10 min timeline. The well-established commercially available surfactant **SLES** was also measured and showed comparable results. These data suggest that a commercial standard foam performance was achieved with these biobased surfactants containing *N*-methylalkyl chains (R_1_ = Me). Furthermore, a compound can be classified as a surfactant if it decreases the air–water interfacial force and lowers the surface tension of the system. The surface tension and corresponding critical micelle concentration (CMC) of the synthesized surfactants were measured using a tensiometer (Table 3 and Figure S1, Page S10). The l-Ara-based compounds **Ara1.8**, **Ara1.10**, and **Ara1.12** had a lower surface tension (31.4, 28.7, 33.7, and 27.8 mN/m) than that of **SLES** (33.8 mN/m). In addition, the CMC of these surfactants was comparable to that of **SLES**, indicating that with low concentrations between 0.01 and 0.25%, micelles are formed. These findings demonstrate that these molecules display suitable properties to be used as surface-active agents. Likewise, the anionic surfactants derived from d-GalA, **GalA1.8, GalA1.10**, and **GalA1.12** reduced the surface tension to 32.1, 32.9, and 33.9 mN/m at a CMC of 1.15, 0.06, and 0.1%, respectively, similar to the commercial surfactant. Interestingly, anionic surfactants **GalA1.12** and **SLES**, both with a 12-carbon chain, had very similar foamability and surface tension performance. This would suggest that a potential application as a wetting agent could be interesting for these compounds. Finally, we compared the performance of the SBP-derived surfactants to Glucopure, a commercially available biobased surfactant based on glucose. Performance was very similar in terms of the solubility, foamability, surface tension, and CMC.

In addition to their physicochemical properties, other important properties were determined to investigate the best possible application of the synthesized surfactants. To this end, we measured the stability of oil/water emulsions and tested the irritability and the biodegradability of the most promising surfactants.

**Figure 2:**
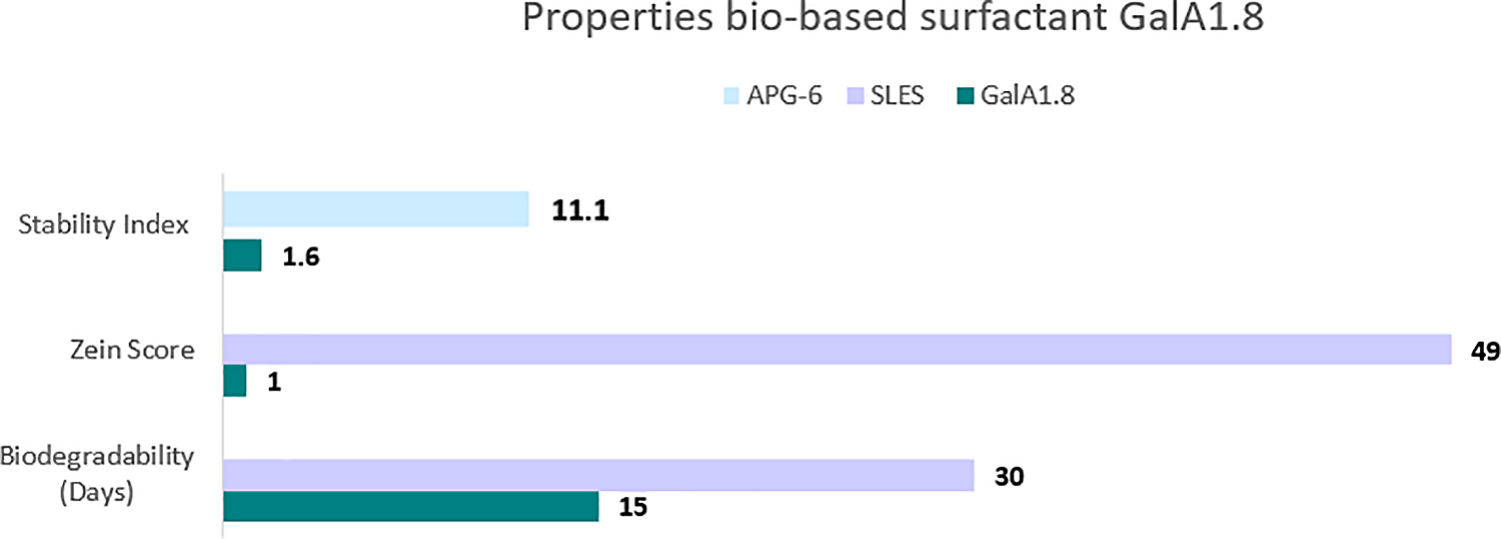
Properties of the biobased surfactant **GalA1.8**, Turbiscan stability index at 30 min, zein irritation test with maximal irritation standard 100 of SDS, and biodegradability in days.

The potential of the surfactants to act as emulsifiers and create stable oil/water emulsions was determined using the Turbiscan stability index (TSI). The Turbiscan was used to measure the transmittance and backscattering of pulsed near-infrared light (880 nm) to monitor the stability of emulsions, which are then expressed as the TSI, with a low TSI score referring to a stable emulsion (see the Supporting Information (SI) for experimental details). To this end, detergency and emulsion properties of the new surface-active agents were determined using the TurbiscanLab protocols (Table S1 and Page S11). Emulsions consisting of 6% oil, 1% surfactant, and 93% H_2_O were prepared. The commercially available surfactant alkylpolyglucoside with a six-carbon chain (**APG-6**) is a well-known oil-in-water emulsifier and was used as a reference. The emulsion with **APG-6** had a TSI score of 11.1. The sample of **GalA1.8** showed more stability with a TSI of 1.6 (Figure 2, upper row). This means that very stable oil-in-water emulsions can be made with **GalA1.8**. Due to the high foamability of surfactants **Ara1.12**, **GalA.12**, and **SLES**, we were unable to measure the emulsifying properties for these compounds.

To investigate if the prepared surfactant may be compatible with a personal care application, the skin irritation potential was investigated using zein solubilization tests (see Page S12 for experimental details). Zein is a protein found in corn and is an accepted mimic of skin proteins. The more zein is solubilized by the surfactant, the harsher the potential to irritate and dry the human skin. Notably, it is an indication of its harshness to the skin and allows a comparison of our surfactants to some commercially available surfactants, even though in vivo testing is always the most ideal for measuring mildness. **SDS** and **SLES** are commercial surfactants and known to be harsh on the skin.^34^ Hence, SDS is set as a default of 100 to compare with the potentials of other surfactants, which are all executed in Duplo (Table S2 and Page S12). Moreover, the surfactants were measured at different pH values, a pH of 4–5, 7, and at its original pH as is. **SLES** showed a skin irritation potential value of 49 at a pH of 7, thus half as irritating as SDS. In contrast, our biobased surfactants all have very low values between 0 and 3 (Table S2). For example, **GalA1.8** had a zein score of 1 at pH 7 (Figure 2, middle row). This suggests that the synthesized surfactants derived from SBP monosaccharides d-GalA and l-Ara are potentially mild for human skin. It is clear from the results that the petrochemically derived surfactants **SDS** and **SLES** are much more irritating for the skin than these biobased compounds. This difference may be a result of the difference in anionic headgroup (carboxylic acid vs sulfate) or the difference in solubility of the two surfactants.

The biodegradation of the novel surfactant **GalA1.8** was examined (see Page S13 for experimental details). Biodegradation is important in order to prevent pollution of the environment. The commercially available surfactant **SLES** has a primary degradation of 94% in 30 days,^35^ depending on the initial concentration, using the Organization for Economic Cooperation and Development (OECD) 301 procedure.^36^ This procedure was used to test **GalA1.8** resulting in a faster biodegradability (Figure 2, bottom row). The compound was added to an inoculate of activated sludge from a municipal wastewater treatment plant (WWTP), including a blank control (no compound), a positive control (inoculum and sodium acetate), and controls for abiotic degradation (compound in medium), adsorption (compound with sterilized inoculum), and toxicity (inoculum with compound and sodium acetate). HPLC/MS analysis showed that 90% of the intact **GalA1.8** was consumed within 15 days (Figure S2 and Page S14) and was followed by CO_2_ production (Figure S3 and Page S14). Furthermore, the condition to evaluate the toxicity of **GalA1.8** on the inoculum revealed that CO_2_ production from acetate was not inhibited by **GalA1.8**, suggesting that the compound was not significantly toxic to the inoculum.

The results mentioned above have revealed the potential applications of the synthesized surfactants, particularly those with alkyl chains of eight carbons or more, as effective cleaning, emulsifying, and foaming agents. The shorter chain derivatives **Ara1.6** and **GalA1.6** are not suitable for application as a surfactant because they do not foam and form stable oil-in-water emulsions. We reasoned that due to their high solubility, they may act as a hydrotrope, compounds that enhance the solubility and reduce the viscosity of other organic substances in water.^37,38^ One well-known example is sodium xylene sulfonate (SXS), a commercially available hydrotrope.^37,39^ To evaluate the hydrotropic behavior of **Ara1.6** and **GalA1.6** against SXS as a benchmark, we conducted experiments using a hydrophobic dye Disperse Red 13 (DR-13) dissolved in water. DR-13 is insoluble in water, and hence, the solution has a low absorption at 525 nm (see Page S15 for details). Upon the increased solubilization of DR-13 in water by the addition of a hydrotrope, the solution absorption will also increase, which was detected using a ultraviolet–visible (UV–vis) spectrophotometer at a wavelength of 525 nm.^39^ The obtained absorbance (Abs) values for DR-13, in relation to the molar concentration of **Ara1.6**, **GalA1.6**, SXS, and the cosolvent acetone, are plotted in Figure S4 (Page S15). SXS exhibited an Abs of 1866 L mol^–1^ cm^–1^ at a concentration of 0.9 M (40 wt %), indicating that the dye could be dissolved with a low concentration of SXS. Acetone, as a cosolvent, showed the same solubilization potential (Abs at 1829), however, only at high concentrations. Our surfactant **Ara1.6** demonstrated significant solubilization capabilities at a very low concentration of 0.25 M with an Abs at 1854. This result suggests that **Ara1.6** can be effectively employed as a hydrotrope in various applications such as improving the solubility of less soluble surfactants in cosmetic formulations. To exclude pH effects on the solubilization of the DR-13 dye upon addition of the **Ara1.6** compound, the experiment was also carried out in a buffer, which led to a very similar performance to SXS. Finally, we utilized **Ara1.6** to boost the solubility of **Ara 1.10** (Figure S5 and page S16). Addition of **Ara1.6** effectively enhanced the solubility of a 1% solution of **Ara1.10**.

## Conclusions

A simple method to prepare biobased surfactants derived from sugar beet pulp (SBP) monosaccharides l-Ara and d-GalA was developed. By employing one-pot reductive amination, a variety of surfactants with different alkyl chain lengths and methyl modifications was synthesized. We optimized the reaction conditions to achieve high yields and easy purification, eliminating the need for excess reagents and using (bio)ethanol as the solvent. Furthermore, we conducted a comprehensive evaluation of various surfactant properties, including solubility, foamability, surface tension, emulsion stability, skin irritation, and biodegradability. Remarkably, the newly synthesized methylated biobased compounds from the **Ara1.X** and **GalA1.X** series exhibited increased solubility compared to their nonmethylated counterparts (R_1_ = H). Additionally, surfactants containing octyl, decyl, and dodecyl alkyl chains, like **GalA1.8**, displayed foamability and surface tension comparable to those of SLES, a commonly used commercial surfactant. On the other hand, the study demonstrated the effectiveness of the short six-alkyl chain-containing surfactant **Ara1.6** as a hydrotrope. These findings demonstrate the promising potential of SBP-derived molecules, which not only exhibit nonirritating properties but also demonstrate comparable or improved performance compared to existing commercially available molecules. By employing a sustainable hydrogenation process to generate the key *N*-methylalkyl (R_1_ = Me) compounds, we have successfully synthesized biobased surfactants with interesting properties for various ecofriendly applications. Further scale up and application testing are needed to identify the ultimate application of the developed molecules.

## Supporting Information Available

Chemicals; instruments; setups; experimental data with synthetic procedures and analyses for each synthesized compound, ^1^H NMR and ^13^C NMR spectra of products; surface tension; Turbiscan stability index data; zein scores; surface tension graphs; biodegradability data; and hydrotrope data (PDF)

## References

[ref1] Wang Z.; Yao S.; Song K.; et al. A bio-based benzoxazine surfactant from amino acids. Green Chem. 2020, 22, 3481–3488. 10.1039/D0GC00218F.

[ref2] Xu Y.; Hanna M. A.; Isom L. Green” chemicals from renewable agricultural biomass-a mini review. Open Agric. J. 2008, 2, 54–61. 10.2174/1874331500802010054.

[ref3] Méline T.; Muzard M.; Deleu M.; et al. d-Xylose and l-arabinose laurate esters: Enzymatic synthesis, characterization and physico-chemical properties. Enzyme Microb. Technol. 2018, 112, 14–21. 10.1016/j.enzmictec.2018.01.008. 29499775

[ref4] Le Guenic S.; Chaveriat L.; Lequart V.; Joly N.; Martin P. Renewable surfactants for biochemical applications and nanotechnology. J. Surfactants Deterg. 2019, 22, 5–21. 10.1002/jsde.12216.

[ref5] Cabeza C.; Gaffey J.; Hatvani N. Potential of Biomass Sidestreams for a Sustainable Biobased Economy; Steinbeis-Edition, 2019; pp 70–73.

[ref6] Di Donatoa P.; Polia A.; Taurisanoa V.; Nicolausa B. Polysaccharides: Applications in Biology and Biotechnology/Polysaccharides from Bioagro-Waste New Biomolecules-Life. In Polysaccharides; Springer, 2014; pp 1–29.

[ref7] van Engelen G.; Raaijmakers H. Harvesting the sun. Specialty Chem. Mag. 2012, 24–25.

[ref8] Farias C. B. B.; Almeida F. C.; Silva I. A.; et al. Production of green surfactants: Market prospects. Electron. J. Biotechnol. 2021, 51, 28–39. 10.1016/j.ejbt.2021.02.002.

[ref9] Finkenstadt V. L. A review on the complete utilization of the sugarbeet. Sugar Tech 2014, 16, 339–346. 10.1007/s12355-013-0285-y.

[ref10] Schäfer D.; Schmitz K.; Weuster-Botz D.; Benz J. P. Comparative evaluation of Aspergillus niger strains for endogenous pectin-depolymerization capacity and suitability for d-galacturonic acid production. Bioprocess Biosyst. Eng. 2020, 43, 1549–1560. 10.1007/s00449-020-02347-z. 32328731 PMC7378126

[ref11] Ward D. P.; Hewitson P.; Cárdenas-Fernández M.; et al. Centrifugal partition chromatography in a biorefinery context: Optimisation and scale-up of monosaccharide fractionation from hydrolysed sugar beet pulp. J. Chromatogr A 2017, 1497, 56–63. 10.1016/j.chroma.2017.03.003. 28366567

[ref12] Leijdekkers A. G. M.; Bink J. P. M.; Geutjes S.; Schols H. A.; Gruppen H. Enzymatic saccharification of sugar beet pulp for the production of galacturonic acid and arabinose, a study on the impact of the formation of recalcitrant oligosaccharides. Bioresour. Technol. 2013, 128, 518–525. 10.1016/j.biortech.2012.10.126. 23202377

[ref13] Lichtenthaler F. W.; Mondel S. Perspectives in the use of low molecular weight carbohydrates as organic raw materials. Pure Appl. Chem. 1997, 69, 1853–1866. 10.1351/pac199769091853.

[ref14] Shaban S. M.; Kang J.; Kim D.-H. Surfactants: Recent advances and their applications. Composites Commun. 2020, 22, 100537 10.1016/j.coco.2020.100537.

[ref15] Corazza M.; Lauriola M.; Zappaterra M.; Bianchi A.; Virgili A. Surfactants, skin cleansing protagonists. J. Eur. Acad. Dermatol. Venereol. 2010, 24, 1–6. 10.1111/j.1468-3083.2009.03349.x. 19614860

[ref16] Eras-Muñoz E.; Farré A.; Sánchez A.; Font X.; Gea T. Microbial biosurfactants: a review of recent environmental applications. Bioengineered 2022, 13, 12365–12391. 10.1080/21655979.2022.2074621. 35674010 PMC9275870

[ref17] Foley P.; Pour A. K.; Beach E. S.; Beach E. S.; Zimmerman J. B. Derivation and synthesis of renewable surfactants. Chem. Soc. Rev. 2012, 41, 1499–1518. 10.1039/C1CS15217C. 22006024

[ref18] Klug P.; Mildner C. Use of N-methyl-N-acylglucamines as solubilizers. U.S. Patent US10,813,862, 2020.

[ref19] Gaudin T.; Lu H.; Fayet G.; et al. Impact of the chemical structure on amphiphilic properties of sugar-based surfactants: A literature overview. Adv. Colloid Interface Sci. 2019, 270, 87–100. 10.1016/j.cis.2019.06.003. 31200263

[ref20] Wang L.; Queneau Y. Green Chemistry and Chemical Engineering; Springer: New York, 2019; pp 349–383.

[ref21] Bois R.; Abdellahi B.; Mika B.; et al. Physicochemical, foaming and biological properties of lowly irritant anionic sugar-based surfactants. Colloids Surf., A 2020, 607, 125525 10.1016/j.colsurfa.2020.125525.

[ref22] Ranoux A.; Raaijmakers H.; Lazeroms R.; Van der Klis F.; Van Es D. Surface active compound based on polyhydroxy acids. EP Patent EP3530646A1, 2019.

[ref23] Connor D. S.; Scheibel J. J.; Severson R. G. Preparation of polyhydroxy fatty acid amides in the presence of solvents. US Patent US5,194,639, 1993.

[ref24] Cho J. E.; Sim D.; Kim Y.; et al. Selective syntheses and properties of anionic surfactants derived from isosorbide. J. Surfactants Deterg. 2018, 21, 817–826. 10.1002/jsde.12182.

[ref25] Czajka A.; Hazell G.; Eastoe J. Surfactants at the design limit. Langmuir 2015, 31, 8205–8217. 10.1021/acs.langmuir.5b00336. 25797065

[ref26] García-Ortiz A.; Vidal J. D.; Climent M. J.; et al. Chemicals from biomass: selective synthesis of N-substituted furfuryl amines by the one-pot direct reductive amination of furanic aldehydes. ACS Sustainable Chem. Eng. 2019, 7, 6243–6250. 10.1021/acssuschemeng.8b06631.

[ref27] Afanasyev O. I.; Kuchuk E.; Usanov D. L.; Chusov D. Reductive amination in the synthesis of pharmaceuticals. Chem. Rev. 2019, 119, 11857–11911. 10.1021/acs.chemrev.9b00383. 31633341

[ref28] Gildersleeve J. C.; Oyelaran O.; Simpson J. T.; Allred B. Improved procedure for direct coupling of carbohydrates to proteins via reductive amination. Bioconjugate Chem. 2008, 19, 1485–1490. 10.1021/bc800153t. PMC2629553 18597509

[ref29] Cosenza V. A.; Navarro D. A.; Stortz C. A. Usage of α-picoline borane for the reductive amination of carbohydrates. Arkivoc 2011, 2011, 182–194. 10.3998/ark.5550190.0012.716.

[ref30] Mitts E.; Hixon R. The reaction of glucose with some amines. J. Am. Chem. Soc. 1944, 66, 483–486. 10.1021/ja01231a055.

[ref31] Pelckmans M.; Renders T.; Van de Vyver S.; Sels B. Bio-based amines through sustainable heterogeneous catalysis. Green Chem. 2017, 19, 5303–5331. 10.1039/C7GC02299A.

[ref32] Gallas A.; Hanauer J. F.; Seitz H.; Weinelt F. Process for the preparation of N-alkypolyhydroxyalkylamines from monoalkylamine and reducing sugar. US Patent US6,365,778, 2002.

[ref33] Gad Elmawla A.; El-Shattory Y.; El-Hamide H. A. improved manual dishwashing liquid detergent compared to that produced by multinational companies in Egyptian market. Egypt. J. Chem. 2018, 61, 651–659. 10.21608/ejchem.2018.2756.1222.

[ref34] Nunes A.; Marques P.; Marto J.; et al. Sugar Surfactant-Based Shampoos. J. Surfactants Deterg. 2020, 23, 809–819. 10.1002/jsde.12415.

[ref35] Pessel F.; Noirbent G.; Boyère C.; et al. Cascading One-pot Synthesis of Biodegradable Uronic Acid-based Surfactants from Oligoalginates, Semi-Refined Alginates and Crude Brown Seaweeds. Molecules 2023, 28, 5201 10.3390/molecules28135201. 37446863 PMC10343331

[ref36] Caracciolo A. B.; Cardoni M.; Pescatore T.; Patrolecco L. Characteristics and environmental fate of the anionic surfactant sodium lauryl ether sulphate (SLES) used as the main component in foaming agents for mechanized tunnelling. Environ. Pollut. 2017, 226, 94–103. 10.1016/j.envpol.2017.04.008. 28411499

[ref37] Rosen M. J.; Kunjappu J. T. Surfactants and Interfacial Phenomena; John Wiley & Sons, 2012; pp 235–271.

[ref38] Kunz W.; Holmberg K.; Zemb T. Hydrotropes. Curr. Opin. Colloid Interface Sci. 2016, 22, 99–107. 10.1016/j.cocis.2016.03.005.

[ref39] Bauduin P.; Renoncourt A.; Kopf A.; Touraud D.; Kunz W. Unified concept of solubilization in water by hydrotropes and cosolvents. Langmuir 2005, 21, 6769–6775. 10.1021/la050554l. 16008386

