# Supplementary material for: Synthesis and Performance of Biobased Surfactants Prepared by the One-Pot Reductive Amination of l-Arabinose and d-Galacturonic Acid
Source: ACS Sustain Chem Eng. 2023 Nov 1;11(45):16117–23. doi: 10.1021/acssuschemeng.3c03753 (PMC10647919; doi:10.1021/acssuschemeng.3c03753)

Supplementary information

Synthesis and performance of bio-based surfactants prepared by the one-pot reductive amination of L-Arabinose and D-Galacturonic Acid

Laura M. Jansen[a], Kim W. M. van Rijbroek[a], Pieter C. den Bakker[a], Dimphna J. Klaassen-Heshof[b], Wiert J. B. Kolkman[a], Niek Venbrux[a], Vienna Migchielsen[a], Joost Hutzezon[a], Wouter B. Lenferink[c], Sebastian Lücker[c], Adeline Ranoux[b], Harry Raaijmakers[b] and Thomas J. Boltje[a]*

a.) Department of Synthetic organic chemistry, Institute for Molecules and Materials, Heyendaalseweg 135, 6525 AJ, Nijmegen, The Netherlands
b.) Cosun RD&I, Cosun Innovation Center, Kreekweg 1, 4671 VA, Dinteloord, The Netherlands
c.) Department of Microbiology, Faculty of Science, Radboud University, Nijmegen, the Netherlands

* corresponding author: thomas.boltje@ru.nl

This PDF file includes:
Total number of pages: 32

Total number of figures: 21

Total number of tables: 2

General methods

All chemicals used were obtained from commercial suppliers in reagent grade (or higher) and used as received. L-Arabinose and D-Galacturonic Acid monohydrate were obtained from the sugar beet pulp achieved by Cosun. NMR spectra were recorded on a Bruker Avance III 400 MHz, Bruker 500 MHz, JEOL JNM-ECZ500R/S3 SuperCool or RoyalHFX spectrometer and the compounds were assigned using ^{1}H NMR, ^{13}C NMR, COSY, HSQCand HMBC spectra. ^{13}C NMR experiments were measured as APT. Chemical shifts were reported in parts per million (ppm.) relative to reference ($CDCl_3$: ^{1}H: 7.26 ppm. and ^{13}C 77.16 ppm.; CD_3OD: 1H: 3.31 ppm. and 13C 49.00 ppm.; D_2O: 1H 4.79 ppm) NMR data are presented in the following way: chemical shift, multiplicity (s = singlet, bs = broad singlet, d = doublet, t = triplet, dd = doublet of doublets, ddd = doublet of doublet of doublets, dtd = doublet of triplet of doublets h = heptet, m = multiplet and/or multiple resonances) and coupling constants *J* in Hz. Reactions were monitored using TLC F254 (Merck KGaA) using UV absorption detection (254 nm) and by spraying them with ninhydrin or cerium ammonium molybdate stain (Hannesian's stain) followed by charring at 300 °C. Mass spectra were recorded on a JEOL AccuTOF CS JMS-T100CS (ESI) mass spectrometer. Purification by flash column chromatography was executed using silica gel 60 (Merck, 0.040-0.063 mm) or using automatic flash column chromatography on a Biotage Isolera Spektra One using SNAP or Silicycle cartridges (Biotage, 30-100 µm, 60 Å) 4-50 g with 0 – 20% H_2O (1% acetic acid) in ACN as eluent.

Synthetic procedures

General synthetic procedure 1: Reductive amination with $NaBH_4$ (Ara8, GalA8, GalA12)

N-alkylamine (2 eq.) was added dropwise to a solution of monosaccharide (1 eq.) in MeOH. The reaction mixture was stirred for 24 h at 20 ˚C. The resulting mixture was cooled to 0 ˚C and $NaBH_4$ (1.5 eq.) was added stepwise.

General synthetic procedure 2: Reductive amination with Pd/C and *N*-Alkylamines (Ara6, Ara1.6, Ara1.8, Ara10, Ara12, Ara1.12, GalA6, GalA1.6, GalA1.8, GalA10, GalA1.12)

To a solution of L-Ara (1.0 gram, 1 eq.) or D-GalA (1.0 gram, 1.1 eq.) and *N*-alkylamine (1 eq.) in MeOH or EtOH, palladium on activated carbon (0.4 gram, 10 wt% loading) was added under an argon atmosphere. The reaction mixture was bubbled through with hydrogen for 15 min and was left stirring for 48/72/96 h at 20 ˚C or 35 ˚C. Every 24 h, the reaction mixture was additionally bubbled through with hydrogen for 15 min. The resulting mixture was purged of hydrogen by flushing with argon for 15 min.

General synthetic procedure 3: Reductive amination with Pd/C, primary amines and formaldehyde (Ara1.10, GalA1.10)

To a solution of L-Ara (1.0 gram, 1 eq.) or D-GalA.H_2O (1.0 gram, 1.1 eq.) and *N*-alkylamine (1 eq.) in EtOH, palladium on activated carbon (Pd/C) (0.4 gram, 10 wt% loading) was added under an inert atmosphere. The reaction mixture was bubbled through with hydrogen for 15 min and was left stirring for 16 – 96h at 35 ˚C. Every 24 h, the reaction mixture was additionally bubbled through with hydrogen for 15 min. After

completion of the first step, 3 eq. of formaldehyde was added, the reaction mixture was bubbled through with hydrogen for 15 min and was left stirring for 16– 48 h at 35 ˚C. The resulting mixture was purged of hydrogen by flushing with argon for 15 min.

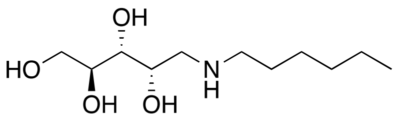

***N*-hexyl-L-arabinonamine (Ara6):** *Via* general synthetic procedure 2 starting from L-Ara (2.0 g, 13.3 mmol). The Pd/C catalyst was filtered off on Celite and washed with H_2O and MeOH. The filtrate was concentrated *in vacuo* to yield **Ara6** (2.5 g, 81%) as a yellow solid. **TLC** (ACN/H_2O, 4/1, v/v): Rf = 0.11; **^{1}H NMR** (500 MHz, MeOD) δ 3.99 (ddd, *J* = 8.1, 4.1, 2.2 Hz, 1H, H-2), 3.79 (ddd, *J* = 10.7, 8.9, 3.2 Hz, 1H, H-5), 3.70 – 3.66 (m, 1H, H-4), 3.64 (ddd, *J* = 11.0, 5.9, 3.2 Hz, 1H, H-5), 3.43 (dd, *J* = 8.0, 2.2 Hz, 1H, H-3), 2.93 – 2.71 (m, 2H, H-1), 2.71 – 2.57 (m, 2H), 1.60 – 1.49 (m, 2H), 1.41 – 1.30 (m, 6H), 0.96 – 0.90 (m, 3H); **^{13}C NMR** (126 MHz, MeOD) δ 72.81 (C-3), 71.68 (C-4), 68.54 (C-2), 63.59 (C-5), 52.24 (C-1), 49.19, 31.50, 29.07, 26.67, 22.25, 12.96; **HRMS** (*m/z*): $[M+H]^+$ calcd for $C_{11}H_{25}NO_4$, 236.18; found, 236.18584

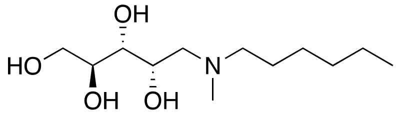

***N*-methylhexyl-L-arabinonamine (Ara1.6)**: *Via* general synthetic procedure 2 starting from L-Ara (2.0 g, 13.3 mmol). The Pd/C catalyst was filtered off on Celite and washed with MeOH. The filtrate was concentrated *in vacuo* to yield **Ara1.6** (3.1 g, 94%) as a white solid. **TLC** (ACN/H_2O, 4/1, v/v): Rf = 0.11; **^{1}H NMR** (500 MHz, MeOD) δ 4.00 (ddd, *J* = 7.4, 4.9, 2.2 Hz, 1H, H-2), 3.81 (dd, *J* = 11.0, 3.4 Hz, 1H, H-5), 3.71 – 3.66 (m, 1H, H-4), 3.66 (m, 1H, H-5), 3.42 (dd, *J* = 8.2, 2.3 Hz, 1H, H-3), 2.66 (dd, *J* = 12.9, 7.5 Hz, 1H, H-1), 2.57 – 2.39 (m, 3H, H-1), 2.33 (s, 3H), 1.58 – 1.48 (m, 2H), 1.41 – 1.30 (m, 6H), 0.97 – 0.89 (m, 3H); **^{13}C NMR** ^{13}C NMR (126 MHz, MeOD) δ 72.77 (C-3), 71.77 (C-4), 67.23 (C-2), 63.68 (C-5), 60.71 (C-1), 58.13, 41.63, 31.54, 26.84, 26.49, 22.28, 12.98; **HRMS** (*m/z*): $[M+Na]^+$ calcd for $C_{12}H_{27}NO_4$, 272.18; found, 272.18291

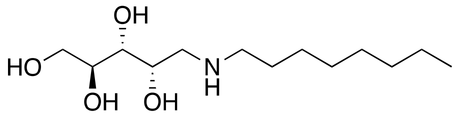

***N*-octyl-L-arabinonamine (Ara8):** *Via* general synthetic procedure 2 starting from L-Ara (2.0 g, 13.3 mmol). The Pd/C catalyst was filtered off on Celite and washed with MeOH. The filtrate was concentrated *in vacuo* to yield **Ara8** (2.94 g, 85%) as a white solid. **TLC** (ACN/H_2O, 4/1, v/v): Rf = 0.12; **^{1}H NMR** (500 MHz, D_2O) δ 3.90 (ddd, J = 7.1, 4.0, 2.2 Hz, 1H, H-2), 3.72 (dd, J = 10.9, 3.4 Hz, 1H, H-5a), 3.61 – 3.52 (m, 2H, H-4, H-5b), 3.35 (dd, J = 7.8, 2.2 Hz, 1H, H-3), 2.74 (dd, J = 12.3, 8.1 Hz, 1H), 2.57 (dddd, J = 38.5, 18.9, 11.9, 7.7 Hz, 3H), 1.47 (p, J = 7.0 Hz, 2H), 1.34 – 1.15 (m, 10H), 0.84 (t, J = 6.6 Hz, 3H); **^{13}C NMR** (126 MHz, MeOD) δ

74.32 (C-3), 73.17 (C-4), 70.06 (C-2), 65.08 (C-5), 53.77 (C-1), 50.70, 33.07, 30.71, 30.66, 30.46, 28.49, 23.79, 14.48.; **HRMS** (*m/z*): $[M+Na]^+$ calcd for $C_{13}H_{29}NO_4$, 286.20; found, 286.19846

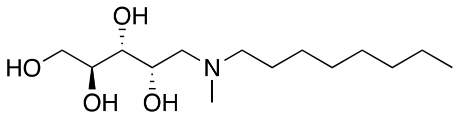

***N*-methyloctyl-L-arabinoamine (Ara1.8)**: *Via* general synthetic procedure 2 starting from L-Ara (2.0 g, 13.3 mmol). The Pd/C catalyst was filtered off on Celite and washed with MeOH. The filtrate was concentrated *in vacuo* to yield **Ara1.8** (3.3 g, 98%) as a yellow solid. **TLC** (ACN/H_2O, 4/1, v/v): Rf = 0.39; **^{1}H NMR** (500 MHz, MeOD) δ 4.01 (ddd, *J* = 7.3, 4.9, 2.3 Hz, 1H, H-2), 3.81 (dd, *J* = 11.0, 3.4 Hz, 1H, H-5), 3.69 (ddd, *J* = 8.2, 5.9, 3.4 Hz, 1H, H-4), 3.64 (dd, *J* = 11.0, 5.8 Hz, 1H, H-5), 3.42 (dd, *J* = 8.2, 2.2 Hz, 1H, H-3), 2.67 (dd, *J* = 12.9, 7.5 Hz, 1H, H-1), 2.58 – 2.40 (m, 3H, H-1), 2.34 (s, 3H), 1.58 – 1.49 (m, 2H), 1.40 – 1.28 (m, 10H), 0.95 – 0.88 (m, 3H); **^{13}C NMR** (126 MHz, MeOD) δ 72.75 (C-3), 71.76 (C-4), 67.19 (C-2), 63.68 (C-5), 60.67 (C-1), 58.11, 41.61, 31.61, 29.25, 29.01, 27.16, 26.47, 22.31, 13.02; **HRMS** (*m/z*): $[M+H]^+$ calcd for $C_{14}H_{31}NO_4$, 278.23; found, 278.23295

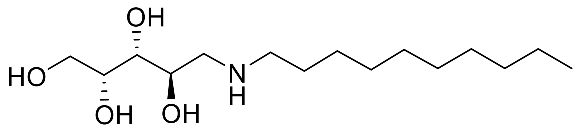

***N*-decyl-L-arabinonamine (Ara10):** *Via* general synthetic procedure 2 starting from L-Ara (2.0 g, 13.3 mmol). The Pd/C catalyst was filtered off over Celite and washed with MeOH. The filtrate was concentrated *in vacuo* and subsequently washed with ethyl acetate. Acetone was added and the product precipitated as a white powder. Filtration was used to yield **Ara10** (283 mg, 7%) as an off-white powder. **TLC** (ACN/H_2O, 4/1, v/v): Rf = 0.67; **^{1}H NMR** (500 MHz, MeOD) δ 3.96 (ddd, *J* = 8.2, 4.1, 2.2 Hz, 1H, H-2), 3.80 – 3.57 (m, 3H, H-4, H-5), 3.39 (dd, *J* = 8.0, 2.2 Hz, 1H, H3), 2.83 – 2.69 (m, 1H, H-1), 2.68 – 2.54 (m 3H, H-1, CH_2), 1.51 (p, *J* = 7.1 Hz, 2), 1.35 – 1.20 (m, 14H), 0.87 (t, *J* = 6.8 Hz, 2H). **^{13}C NMR** (126 MHz, MeOD) δ 72.74 (C-3), 71.69 (C-4), 68.45 (C-2), 63.61 (C-5), 52.16 (C-1), 29.33, 29.27, 28.95, 26.99, 22.38, 13.11. **HRMS** (*m/z*): $[M+H]^+$ calcd for $C_{15}H_{33}NO_4$, 292,24; found, 292.24982

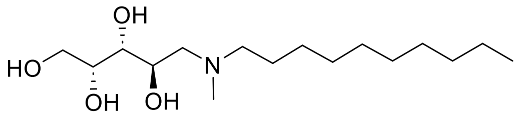

***N*-methyldecyl-L-arabinosamine (Ara1.10)**: *Via* general procedure 3 using an inert argon atmosphere in EtOH, starting from L-Ara (1g, 7.6 mmol). The Pd/C catalyst was filtered off over Celite and washed with EtOH. The filtrate was concentrated *in vacuo* to yield compound **Ara1.10** (1.998 g, 98%) as an off-white solid. **TLC** (ACN/H_2O(1%AA), 4/1, v/v): R_f = 0.15 **^{1}H NMR** (500 MHz, D_2O) δ 4.16 (dt, J = 9.9, 2.5 Hz, 1H, H-2), 3.73 (dd, J = 11.7, 2.9 Hz, 1H, H-5a), 3.62 (ddd, J = 8.7, 5.8, 2.8 Hz, 1H, H-4), 3.55 (dd, J = 11.7, 5.9 Hz, 1H, H-5b), 3.35 (dd, J = 8.7, 1.8 Hz, 1H, H-3), 3.14 (dd, J = 13.2, 10.2 Hz, 1H, H-1b), 2.95 (ddd, J = 16.3, 11.3, 4.3 Hz, 3H, H-1a, CH_2), 2.68 (s, 3H), 1.61 – 1.53 (m, 2H), 1.22 – 1.15 (m, 14H), 0.76 (s, 3H). **^{13}C NMR** (126 MHz,D_2O) δ 71.30 (C-3), 70.70

(C-4), 64.91 (C-2), 62.97 (C-5), 58.78 (C-1), 57.22, 40.76, 31.56, 29.12, 29.06, 28.93, 28.78, 26.32, 23.99, 22.35, 13.67. **HRMS** *(m/z)*: $[M+H]^+$ calculated for $C_{16}H_{35}NO_4$, 306.25661; found, 306.26254

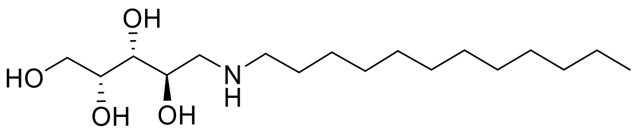

***N*-dodecyl-L-arabinoamine (Ara12)**: *Via* general synthetic procedure 2 starting from L-Ara (2.01 g, 13.4 mmol). The Pd/C catalyst was filtered off on Celite and washed with MeOH. The filtrate was concentrated *in vacuo* to yield **Ara12**(1.155 g, 27%) as a yellow solid. **TLC** (ACN/H_2O, 4/1, v/v): Rf = 0.56; **^{1}H NMR** (500 MHz, MeOD) δ 3.96 (ddd, J = 8.2, 4.1, 2.2 Hz, 1H, H-2), 3.76 (dd, J = 10.8, 3.2 Hz, 1H, H-5a), 3.64 – 3.57 (m, 2H, H-5b, H-4), 3.38 (dd, J = 7.9, 2.2 Hz, 1H, H-3), 2.86 – 2.56 (m, 2H, H-1), 2.71 – 2.50 (m, 2H), 1.56 – 1.46 (m, 1H), 1.36 – 1.19 (m, 18H), 0.87 (t, J = 6.9 Hz, 1H).**^{13}C NMR (**126 MHz, MeOD) δ 74.40 (C3), 72.76 (C4), 70.28 (C2), 63.40 (C5), 51.17 (C1), 49.21, 31.73, 29.44, 29.41, 29.39, 29.35, 29.26, 29.11, 29.04, 27.02, 22.39, 13.15. **HRMS** (*m/z*): $[M+H]^+$ calcd for $C_{17}H_{37}NO_4$, 320.27; found, 320.27941

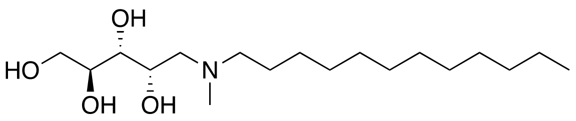

***N*-methyldodecyl-L-arabinoamine (Ara1.12)**: *Via* general synthetic procedure 2 starting from L-Ara (2.0 g, 13.3 mmol). The Pd/C catalyst was filtered off on Celite and washed with 3:1 EtOH/H_2O. The filtrate was concentrated *in vacuo* to yield **Ara1.12** (3.8 g, 85%) as a white solid. **TLC** (ACN/H_2O, 4/1, v/v): Rf = 0.22; **^{1}H NMR** (500 MHz, MeOD) δ 4.00 (ddd, *J* = 7.3, 4.9, 2.3 Hz, 1H, H-2), 3.81 (dd, *J* = 11.0, 3.4 Hz, 1H, H-5), 3.72 – 3.66 (m, 1H, H-4), 3.63 (dd, *J* = 11.0, 5.8 Hz, 1H, H-5), 3.42 (dd, *J* = 8.2, 2.3 Hz, 1H, H-3), 2.65 (d, *J* = 7.5 Hz, 1H, H-1), 2.56 – 2.41 (m, 3H, H-1), 2.33 (s, 3H), 1.53 (p, *J* = 7.1, 6.6 Hz, 2H), 1.32 (d, *J* = 10.9 Hz, 20H), 0.95 – 0.89 (m, 3H); **^{13}C NMR (**126 MHz, MeOD) δ 72.77 (C-3), 71.77 (C-4), 67.22 (C-2), 63.68 (C-5), 60.70 (C-1), 58.13, 41.63, 31.67, 29.38, 29.35, 29.34, 29.29, 29.07, 29.06, 27.17, 26.51, 22.33, 13.03; **HRMS** (*m/z*): $[M+Na]^+$ calcd for $C_{18}H_{39}NO_4$, 356.28; found, 356.27714

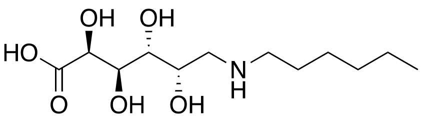

***N*-hexyl-D-galactaricamine (GalA6)**: *Via* general synthetic procedure 2 starting from D-GalA (2.0 g, 10.3 mmol). The Pd/C catalyst was filtered off on Celite and washed with H_2O. The filtrate was concentrated *in vacuo* to yield **GalA6** (2.4 g, 92%) as a white solid. **TLC** (ACN/H_2O, 4/1, v/v): Rf = 0.25; **^{1}H NMR** (500 MHz, D_2O) δ 4.21 – 4.12 (m, 2H, H-2, H-5), 3.89 (dt, *J* = 9.6, 1.2 Hz, 1H, H-4), 3.51 (dt, *J* = 9.7, 1.2 Hz, 1H, H-3), 3.25 – 3.11 (m, 2H, H-1), 3.08 – 2.99 (m, 2H), 1.70 – 1.58 (m, 2H), 1.32 (t, *J* = 7.4 Hz, 2H), 1.25 (dt, *J* = 7.1, 3.7 Hz, 4H), 0.81 (tt, *J* = 7.0, 1.6 Hz, 3H); **^{13}C NMR** (126 MHz, D_2O) δ 179.57 (COOH), 71.32 (C-5), 71.21 (C-4), 70.73 (C-3), 65.60 (C-2), 50.43 (C-1), 47.86, 30.41, 25.32, 25.25, 21.69, 13.18; **HRMS** (*m/z*): $[M+Na]^+$ calcd for $C_{12}H_{25}NO_6$, 380.17; found, 380.17554

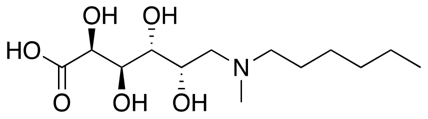

***N*-methylhexyl-D-galactaricamine (GalA1.6)**: *Via* general synthetic procedure 2 starting from D-GalA (2.0 g, 10.3 mmol). The Pd/C catalyst was filtered off on Celite and washed with MeOH. The filtrate was concentrated *in vacuo* to yield **GalA1.6** (2.5 g, 90%) as a white solid. **TLC** (ACN/H_2O, 4/1, v/v): Rf = 0.16; **^{1}H NMR** (500 MHz, MeOD) δ 4.38 – 4.31 (m, 1H, H-2), 4.22 (d, *J* = 1.8 Hz, 1H, H-5), 3.97 (dd, *J* = 9.3, 1.8 Hz, 1H, H-4), 3.50 (dd, *J* = 9.3, 1.4 Hz, 1H, H-3), 3.40 (dd, *J* = 13.3, 10.6 Hz, 1H, H-1), 3.26 – 3.14 (m, 3H, H-1), 2.94 (s, 3H), 1.87 – 1.68 (m, 2H), 1.46 – 1.35 (m, 6H), 0.98 – 0.91 (m, 3H); **^{13}C NMR (**126 MHz, MeOD) δ 178.50 (COOH), 71.64 (C-4), 71.40 (C-3), 71.38 (C-5), 64.61 (C-2), 59.00 (C-1), 56.66, 40.20, 31.06, 25.94, 23.53, 22.11, 12.89; **HRMS** (*m/z*): $[M+Na]^+$ calcd for $C_{13}H_{27}NO_6$, 316.17; found, 316.17365

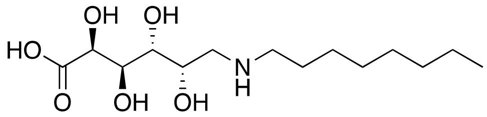

***N*-octyl-D-galactaricamine (GalA8)**: *Via* general synthetic procedure 1 starting from D-GalA (2.0 g, 10.3 mmol). After stirring for 2.5 h with $NaBH_4$, the pH of the reaction mixture was lowered to 1 by dropwise addition of 6 M HCl. The resulting suspension was filtered to yield **GalA8** (2.9 g, 91%) as a white solid. **TLC** (ACN/H_2O, 4/1, v/v): Rf = 0.2; **^{1}H NMR** (500 MHz, D_2O) δ 4.56 (d, *J* = 1.7 Hz, 1H, H-5), 4.23 (ddd, *J* = 9.8, 3.3, 1.5 Hz, 1H, H-2), 4.06 (dd, *J* = 9.7, 1.7 Hz, 1H, H-4), 3.63 (dd, *J* = 9.8, 1.6 Hz, 1H, H-3), 3.30 – 3.17 (m, 2H, H-1), 3.13 – 3.07 (m, 2H), 1.71 (q, *J* = 7.7, 7.3 Hz, 2H), 1.36 – 1.23 (m, 10H), 0.89 – 0.82 (m, 3H); **^{13}C NMR** (126 MHz, D_2O) δ 176.74 (COOH), 70.94 (C-4), 70.18 (C-5), 70.09 (C-3), 65.43 (C-2), 50.38 (C-1), 47.89, 30.97, 28.14, 28.11, 25.65, 25.29, 21.95, 13.36; **HRMS** (*m/z*): $[M+H]^+$ calcd for $C_{14}H_{29}NO_6$, 308.20; found, 308.20733

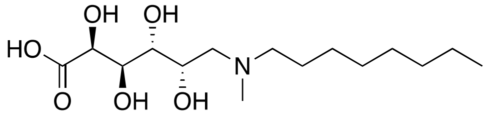

***N*-methyloctyl-D-galactaricamine (GalA1.8)**: *Via* general synthetic procedure 2 starting from D-GalA (10.0 g, 51.5 mmol). The Pd/C catalyst was filtered off on Celite and washed with MeOH and H_2O. The filtrates of the MeOH and H_2O washes were collected in separate flasks and were concentrated *in vacuo* to yield **GalA1.8** (13.2 g, 88%) as an ivory solid. **TLC** (ACN/H_2O, 4/1, v/v): Rf = 0.2; **^{1}H NMR** (500 MHz, D_2O) δ 4.28 (d, *J* = 12.2 Hz, 1H, H-2), 4.18 (t, *J* = 1.2 Hz, 1H, H-5), 3.90 (dt, *J* = 9.8, 1.2 Hz, 1H, H-4), 3.47 (dt, *J* = 9.7, 1.2 Hz, 1H, H-3), 3.40 (d, *J* = 11.1 Hz, 1H, H-1), 3.15 (dd, *J* = 15.7, 12.3 Hz, 3H, H-1), 2.86 (s, 3H), 1.69 (dt, *J* = 15.8, 7.5 Hz, 2H), 1.21 (td, *J* = 8.2, 4.8 Hz, 10H), 0.83 – 0.77 (m, 3H); **^{13}C NMR** (126 MHz, D_2O) δ 179.15 (COOH), 71.34 (C-5), 71.21 (C-4), 70.48 (C-3), 64.02 (C-2), 57.96 (C-1), 50.43, 39.57, 30.96, 28.12, 28.11, 25.62, 23.19, 21.96, 13.37; **HRMS** (*m/z*): $[M+H]^+$ calcd for $C_{15}H_{31}NO_6$, 322.22; found, 322.22274

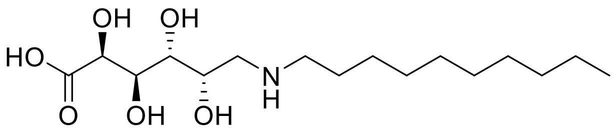

***N*-decyl-D-galactaricamine (GalA10)**: *Via* general synthetic procedure 2 starting from D-GalA (1.0 g, 4.7 mmol). The Pd/C catalyst was filtered off on Celite and washed with MeOH and a basic solution. The filtrate was concentrated *in vacuo* to yield **GalA10** (30 mg, 2%) as a yellow solid. **TLC** (ACN/H_2O, 4/1, v/v): Rf = 0.63; **^{1}H NMR** (500 MHz, MeOD-D_4) δ 4.15 (d, *J* = 5.1 Hz, 1H, H-2), 4.12 (d, *J* = 2.2 Hz, 1H, H-5), 3.86 (d, *J* = 8.8 Hz, 1H, H-4), 3.47 (d, *J* = 9.1 Hz, 1H, H-3), 3.21 – 3.04 (m, 2H, H-1ab), 2.98 (q, *J* = 6.9 Hz, 2H, CH_2), 1.67 (d, *J* = 9.1 Hz, 2H), 1.39 – 1.20 (m, 14H), 0.88 (dd, *J* = 7.7, 5.6 Hz, 3H).**^{13}C NMR** (126 MHz, MeOD-D_4) δ 178.13 (COOH), 71.99 (C-3), 71.67 (C-4), 71.50 (C-2), 66.06 (C-5), 50.93 (C-1), 47.31, 31.71, 29.27, 29.18, 29.08, 28.91, 26.29, 25.85, 22.38, 13.05. **HRMS** (*m/z*):[M+H]$^+$ calcd for $C_{16}H_{33}NO_6$, 336.23; found, 336.23938

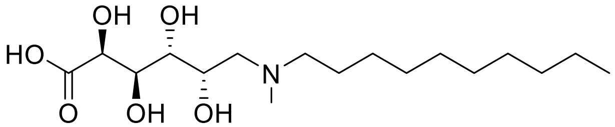

***N*-methyldecyl-D-galactaricamine (GalA1.10):** *Via* general procedure 3 using an inert argon atmosphere in EtOH, starting from D-GalA (2g, 10.30 mmol). The Pd/C catalyst was filtered off over Celite and washed with EtOH. The filtrate was concentrated *in vacuo* and freeze-dried to yield compound **GalA1.10 (**2.39 gr, 73%) as a white powder. **TLC** (ACN/H_2O(1%AA), 4/1, v/v): R_f = 0.22. **^{1}H NMR** (500 MHz, MeOD) δ 4.35 – 4.29 (m, 1H, H5), 4.20 (d, *J* = 1.9 Hz, 1H, H3), 3.94 (dd, *J* = 9.4, 1.9 Hz, 1H, H4), 3.51 – 3.46 (m, 1H, H2), 3.40 (dd, *J* = 13.0, 10.3 Hz, 1H), 3.21 (d, *J* = 11.9 Hz, 2H, H1), 3.17 (d, *J* = 5.2 Hz, 2H), 2.93 (s, 3H), 1.89 – 1.65 (m, 2H), 1.47 – 1.16 (m, 14H), 1.03 – 0.78 (m, 3H).**^{13}C NMR** (126 MHz, MeOD) δ 178,19 (COOH), 71.62 (C4), 71.42 (C2), 71.36 (C3), 64.60 (C5), 56.73 (C1), 59.17, 40.06, 31.70, 29.22, 29.17, 29.02, 28.87, 26.33, 23.66, 22.41, 13.02 **HRMS** *(m/z)*: [M+H]$^+$ calculated for $C_{17}H_{35}NO_6$, 350.24644; found, 350.25369

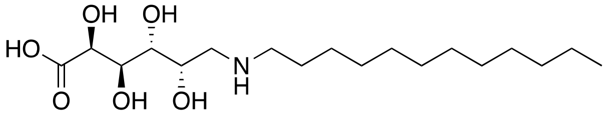

***N*-dodecyl-D-galactaricamine (GalA12)**: *Via* general synthetic procedure 1 starting from D-GalA (2.0 g, 10.3 mmol). After stirring for 2.5 h with $NaBH_4$, a yellow suspension had formed. The resulting suspension was filtered and washed with MeOH to yield **GalA12** (0.1 g, 4%) as a yellow solid. **TLC** (ACN/H_2O, 4/1, v/v): Rf = 0.13; **HRMS** (*m/z*): [M+Na]$^+$ calcd for $C_{18}H_{37}NO_6$, 386.25; found, 386.25232

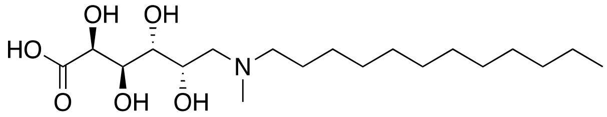

***N*-methyldodecyl-D-galactaricamine (GalA1.12)**: *Via* general synthetic procedure D starting from D-GalA (10.0 g, 51.5 mmol). The Pd/C catalyst was filtered off on Celite. The residue was washed with 3:1 EtOH/H_2O which was collected in a separate flask. The filtrate of the EtOH/H_2O wash was concentrated *in vacuo* to yield **GalA1.12** (15.1 g, 86%) as a white solid. **TLC** (ACN/H_2O, 4/1, v/v): Rf = 0.3; **^{1}H NMR** (500 MHz, EtOD) δ 4.36 (ddd, *J* = 10.7, 2.8, 1.5 Hz, 1H, H-2), 4.21 (d, *J* = 1.6 Hz, 1H, H-5), 3.98 (dd, *J* = 9.7, 1.6 Hz, 1H, H-4), 3.53 (dd, *J* = 9.6, 1.5 Hz, 1H, H-3), 3.44 (dd, *J* = 13.2, 10.7 Hz, 1H, H-1), 3.22 (td, *J* = 12.2, 9.6, 4.4 Hz, 3H, H-1), 2.95 (s, 3H), 1.79 (dp, *J* = 24.0, 9.1, 7.6 Hz, 2H), 1.39 – 1.24 (m, 20H), 0.92 – 0.85 (m, 3H); **^{13}C NMR** (126 MHz, EtOD) δ 178.99 (COOH), 71.33 (C-5), 71.26 (C-4), 70.87 (C-3), 64.32 (C-2), 58.63 (C-1), 40.39, 31.65, 29.38, 29.35, 29.32, 29.29, 29.04, 28.99, 26.34, 23.73, 23.36, 22.34, 13.42.; **HRMS** (*m/z*): $[M+Na]^+$ calcd for $C_{19}H_{39}NO_6$, 400.27; found, 400.26864

Solubilities in water were determined by preparing mixtures of an excess of compound dispersed in 4 ml demi water using a vortex at maximum speed for 1 min to create a saturated solution which was left to settle. 1 mL of the clear solution was taken and concentrated *in vacuo* to yield a solid. The weight of the solid was used to calculate the solubility expressed as a w/w % ratio.

Foam testing of the surfactants were performed using an IKA Ultra-Turrax T25 with an 18G dispersing element. At room temperature, an aqueous solution of 5 mL 0.4% surfactant in a 50 mL measuring cylinder (2.20 cm diameter) was mixed with the Ultra-Turrax for 20 seconds at 8000 rpm. The foam height (volume in mL) was noted for 0, 1, 3, 5, 10, 30, 60 min and 24 hours. 1 eq sodium hydroxide was added to to rGalA1.10 and rGalA1.12 surfactants which displayed poor solubility to increase their solubility.

Surface tension measurements were recorded on a Biolin Scientific Force Tensiometer Sigma 701, equipped with a T107 platinum Wilhelmy plate. Aqueous surfactant solutions, 4 mL of 0.05 – 6% active ingredient, were measured at room temperature using the 'Continuous Wilhelmy plate' method. The average surface tension was plotted against the concentration to derive the critical micelle concentration (CMC), surface tension at critical micelle concentration (γ_{CMC}).

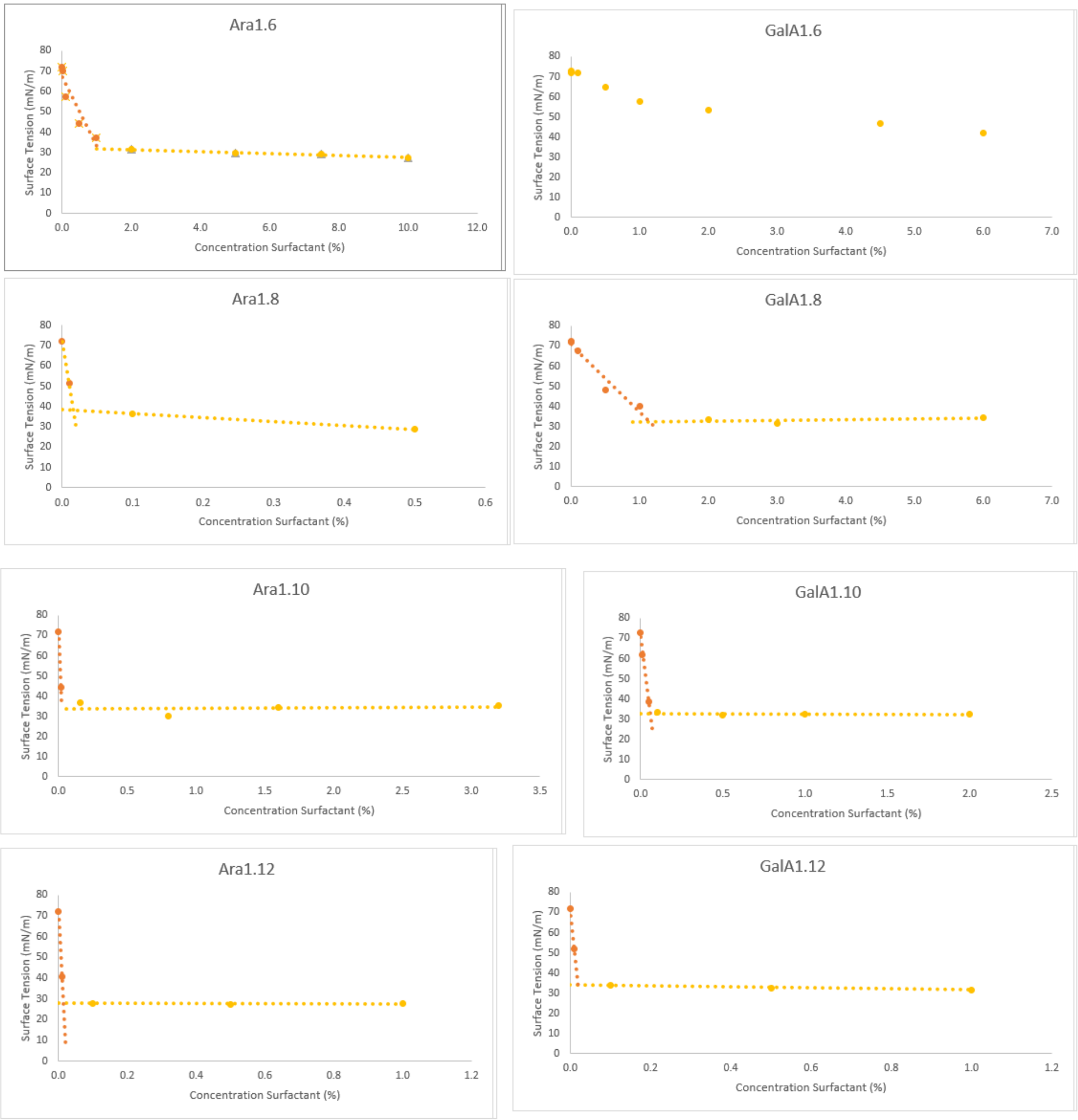

Figure S1: Surface tension graphs at different concentrations (w/w %)

Turbiscan Index Stability (TSI) was determined in emulsions with the newly synthesized surfactants. The emulsions are obtained using a vortex for 60 seconds with the following composition: 6% of sunflower oil, 1% of surfactants and 91% of water. The emulsions are directly analyzed using the Turbiscan by scanning the sample every 30 seconds during 30 minutes. In some cases 20% oil was used to determine the emulsifier property of the surfactants.

Table S1: Turbiscan stability index to determine detergency properties

Compounds	5(min)	10(min)	20(min)	30(min)
Ara1.6 pH5 (6% oil)	1,2	1,6	3,2	6,3
Ara1.8 pH 5 (6% oil)	2,8	3,3	4,3	4,9
GalA1.8 pH 5 (6% oil)	0,8	1,1	1,3	1,6
APG6 pH 5 (6% oil)	5,7	7,8	10	11,1
Ara1.6 (20% oil)	2,1	2,9	3,8	6,1
GalA1.6 (20% oil)	6,4	7,7	9,4	10,3
APG6 (20% oil)	3,9	6,8	10,5	13,3

Zein scores

Zein solubilisation was measured to determine the irritation of the surfactants. Zein is a protein found in corn. It is used as a mimic of skin proteins, the more solubilised by the surfactant, the harsher the potential to irritate and dry the skin. This method is a quick screen but like other methods it has pitfalls. It is indicative and should not be relied on solely to determine other factors that may influence skin irritation. The procedure is 1) Weight 2-2.5 ml Eppendorf's on a balance. 2) Weigh out as accurately as possible 70 mg of Zein. 3) Add to the Eppendorf and reweigh. 4) Make your surfactant dilutions. 5) Add 1 ml of the surfactant dilution to the Eppendorf. 6) Place this on a rotary mixer for 20 mins at 20rpm at RT. 7) Remove and centrifuge for 4 mins at highest speed available on a micro centrifuge 8) Discard the supernatant making sure as much of it possible is removed. 9) Repeat with water, centrifuge and discard supernatant. 10) With the Eppendorf lid open, place at 50 °C overnight to dry (48 hours required). 11) Re-weigh the next day. 12) Calculate the amount of Zein solubilised. Like this mentions, it is indicative but gives some good data to compare surfactants and their harshness to skin. Though, in vivo testing is always the most ideal for measuring mildness.

Table S2: Zein Scores of surfactants

Label	Zein Score
Ara1.6 pH 4-5	1
Ara1.6 pH 7	1
Ara1.6 pH 10.6	1
Ara1.8 pH 4-5	0
Ara1.8 pH 7	1
Ara1.8 pH 9.3	0
Ara1.12 pH 4-5	25
Ara1.12 pH 7.1	0
GalA1.6 pH 7	3
GalA1.6 pH 9.4	2
GalA1.8 pH 7	1
GalA1.8 pH 8.6	0
GalA1.12 pH 4-5	2
GalA1.12 pH 7	0
SLES pH 7	49
SLES pH 11	55
SDS	100

Biodegradability

The biodegradability of GalA1.8 was investigated. The compound was added to an inoculate of activated sludge from a municipal wastewater treatment plant (WWTP). The experiment relies on the basic principles of the 301A DOC Die-Away Test described in the OECD guidelines for readily biodegradable substances. Only a small adjustment was made: instead of measuring in conical flasks but in serum vials closed with butyl rubber stoppers. This allows us to measure the evolution of CO_2 and O_2 over time. In general, the experiment involves the experimental incubation (inoculum and compound), along with a blank control (inoculum no compound), a positive control (inoculum and sodium acetate), and controls for abiotic degradation (compound in medium), adsorption (compound with sterilized inoculum), and toxicity (inoculum with compound and sodium acetate).

Sludge and medium

Activated sludge wat obtained from the wastewater treatment facility in Weurt (Netherlands) from an aerated sludge basin. The basin was operated at 20.7 °C and contained 2.29 g/L total suspended solids (TSS) and 1.04 g/L dissolved oxygen (DO). The fresh sludge was conditioned overnight by bubbling with air at room temperature. Before incubation, the sludge was washed once in mineral medium containing per L ddH_2O: 85 mg KH_2PO_4, 217.5 mg K_2HPO_4, 334 mg Na_2HPO_4*2H_2O, 5 mg NH_4Cl, 27.5 mg $CaCl_2$, 22.5 mg $MgSO_4$*7H_2O, 0.25 mg $FeCl_3$*6H_2O, and 0.4 mg 2Na-EDTA.

Incubations

Incubations were prepared containing approximately 70 mg/L surfactant and 3 ml sludge in a final volume of 30 ml mineral medium in a borosilicate serum vial crimp-sealed with a butyl rubber stopper. In no sludge controls, the sludge was replaced with additional medium. In inactive controls, autoclaved (20 min at 120 °C) sludge was used. In toxicity controls, approximately 135 mg/L sodium acetate was added alongside sludge and surfactant. In no substrate controls, both acetate and surfactant were omitted. In acetate only controls, 135 mg/L sodium acetate was added as the sole carbon source. All incubations were kept at 20 °C shaking 300 rpm throughout the duration of the experiment.

1 ml samples were taken weekly for surfactant quantification and centrifuged 5 min at 20.000 x g. The resulting supernatant was filtered using a 0.2 µm syringe filter and diluted 4:1 with ice-cold 1:1 acetonitrile:methanol and stored at -70 °C until analysis. Calibration curves of the surfactant were prepared by spiking surfactant in the filtered supernatant of the substrate-free controls to account for matrix effects. We measured the calibration curve in both medium and "spent medium" of the inoculum and looked at the "intact" surfactant. LC-MS measurements were performed on a 1290 Infinity II liquid chromatography (LC) system coupled to a 6546 quadrupole time of flight mass spectrometer (Agilent Technologies) according to (1). CO_2 was analyzed by measuring headspace gas on an Agilent 8890A/5977B GC-MS equipped with an Agilent 6 FT Porapak Q 80/10 column.

1. Jansen RS, Mandyoli L, Hughes R, Wakabayashi S, Pinkham JT, Selbach B, Guinn KM, Rubin EJ, Sacchettini JC, Rhee KY. 2020. Aspartate aminotransferase Rv3722c governs aspartate-dependent nitrogen metabolism in Mycobacterium tuberculosis. Nat Commun 11:1–13.

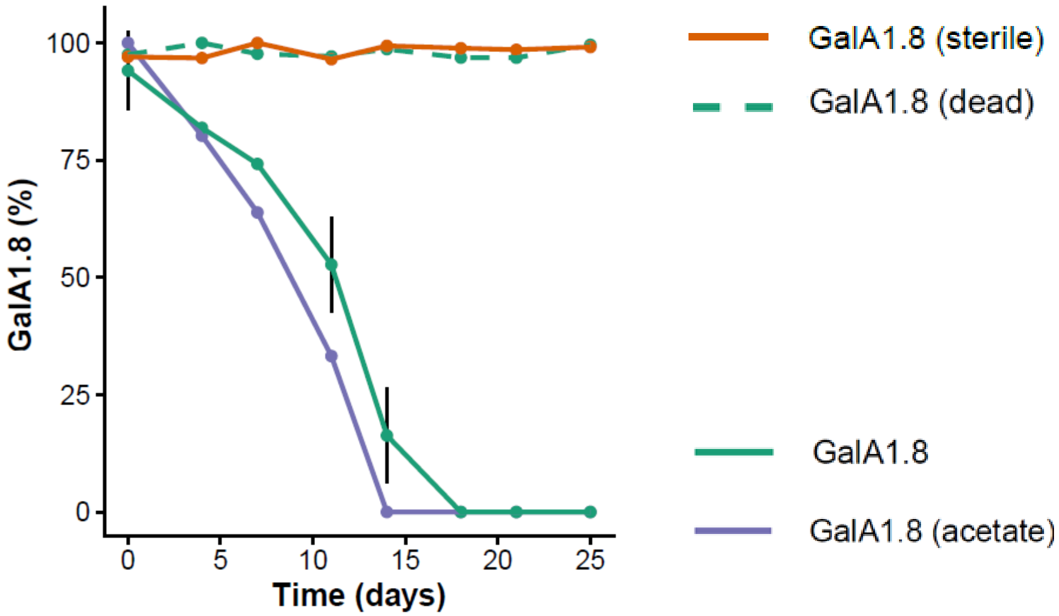

Figure S2: the degradation of the surfactant over days.

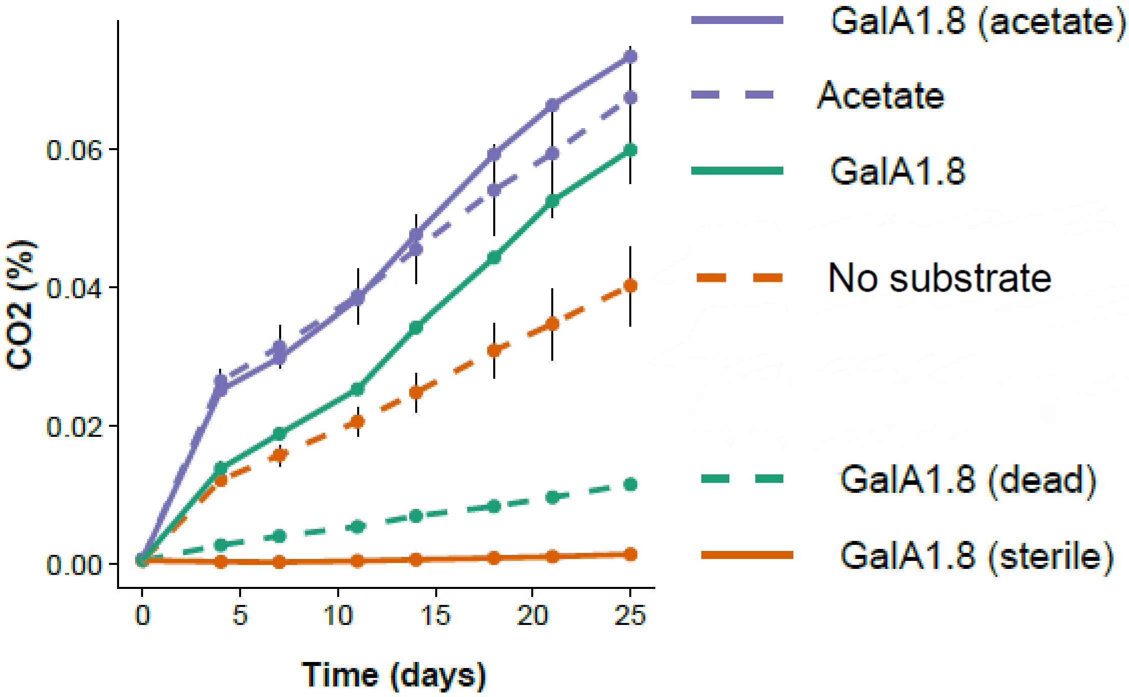

Figure S3: The evolution of CO_2 during degradation

Hydrotrope Data

In the solubilization studies of DR-13, the concentration and amount of DR-13 dissolved in water were measured using a range of different concentrations of hydrotropes. The experiments were performed at room temperature. Specifically, 6 mg of the DR-13 dye was added to each solution, and water was added to make a total volume of 1 ml. The solutions were allowed to equilibrate for 24 hours, and then they were filtered to remove any undissolved excess of DR-13 dye. To quantify the concentration of dissolved DR-13, the absorbance of the solutions was measured at a wavelength of 525 nm, which corresponds to the λmax of the DR-13 dye, where it has its absorption maximum. Before each measurement of a different product, a baseline absorbance was taken using pure water. The hydrotropes were measured in a water solution with a pH as is. In addition, Ara1.6 was measured in a natural buffer solution.

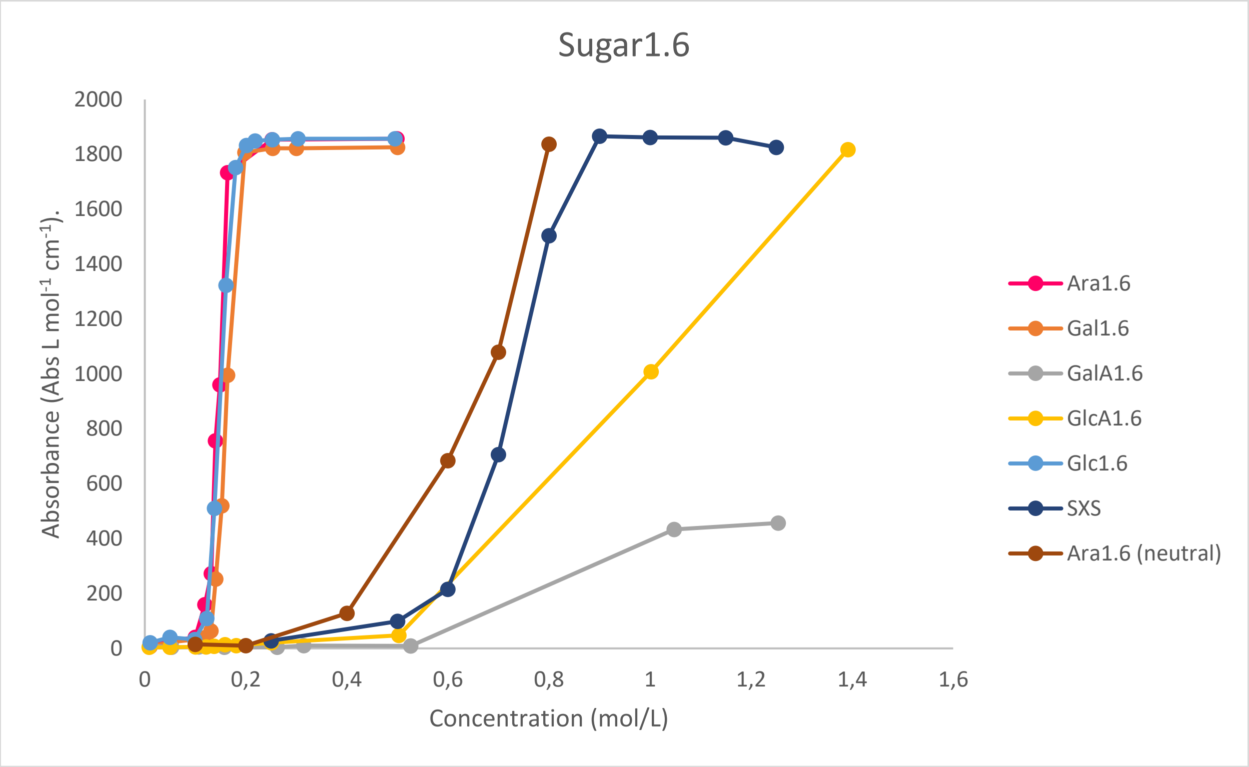

Figure S4: Hydrotrope Data sugars with six carbon chain and commercially available hydrotrope SXS

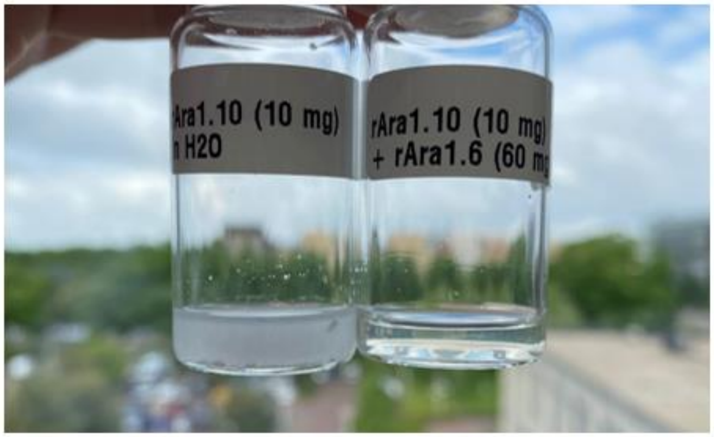

Figure S5: Left a 1% solution of Ara1.10 in water, right the same solution plus 60 mg of Ara1.6

NMR spectra data

Figure S6: NMR data Ara6

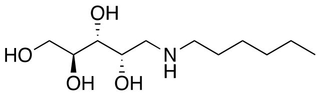

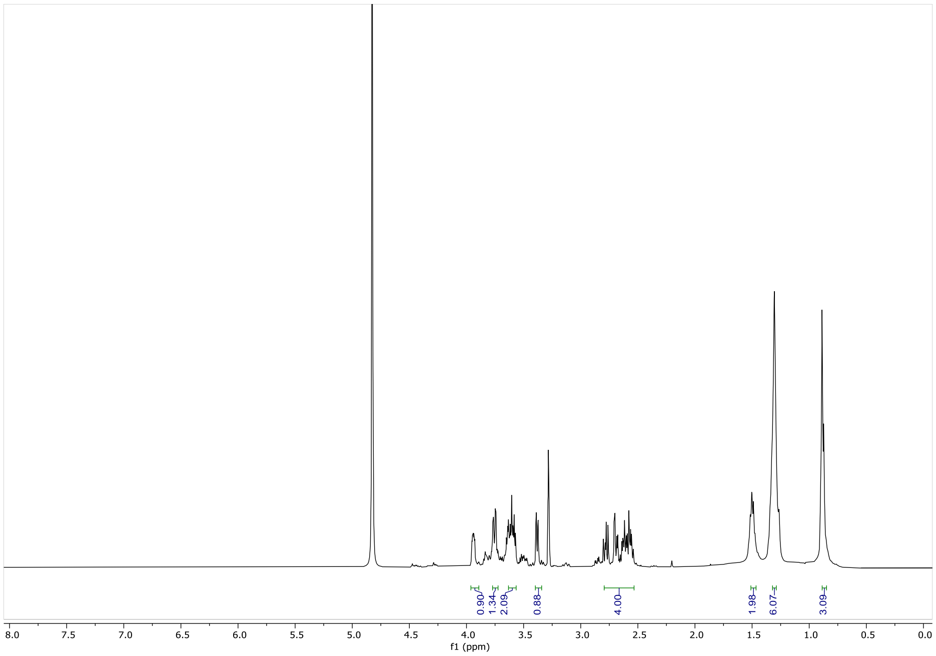

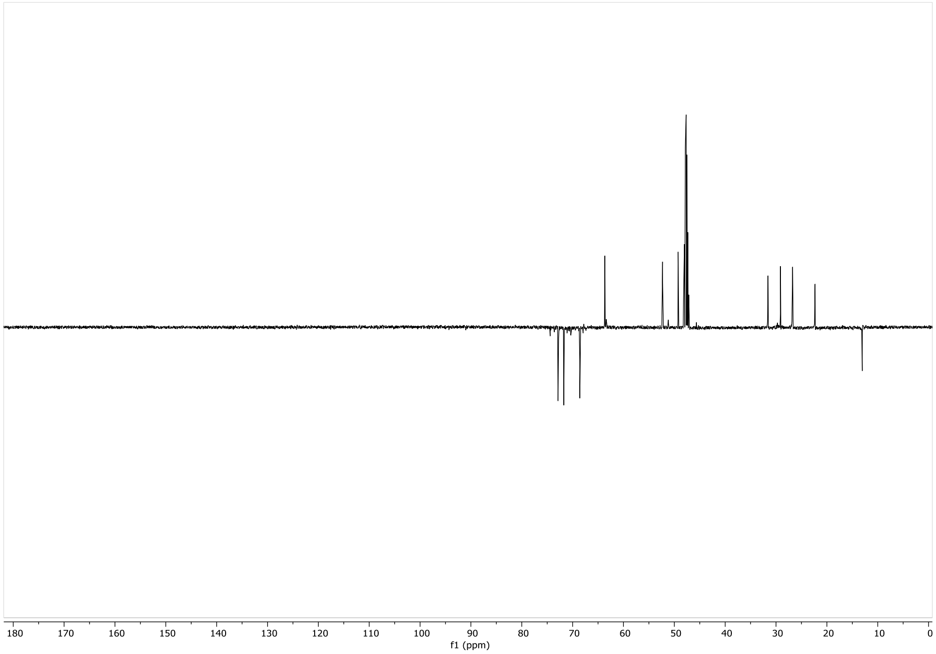

Figure S7: NMR data Ara1.6

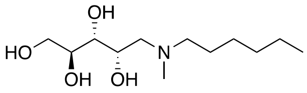

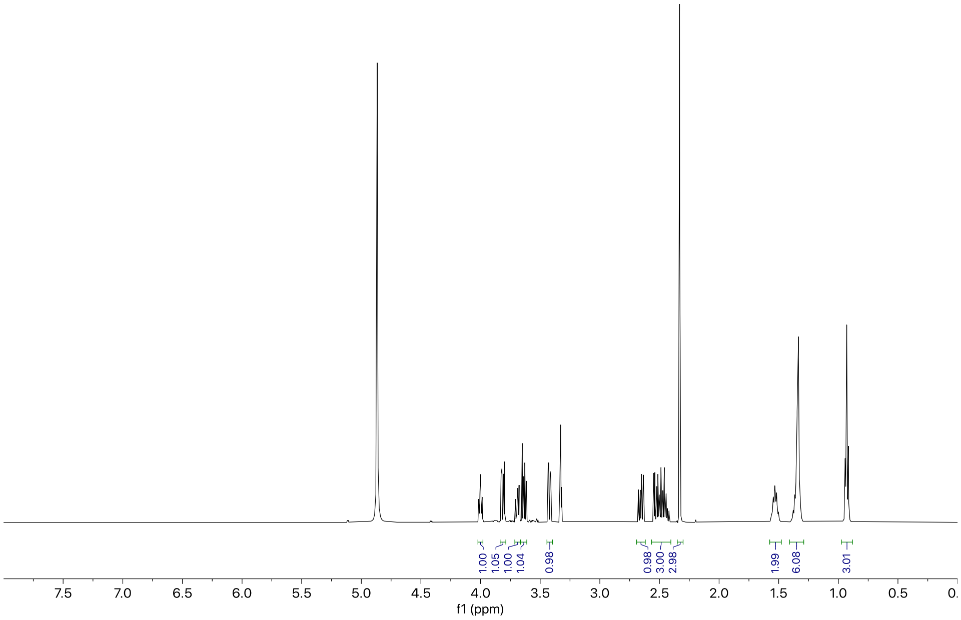

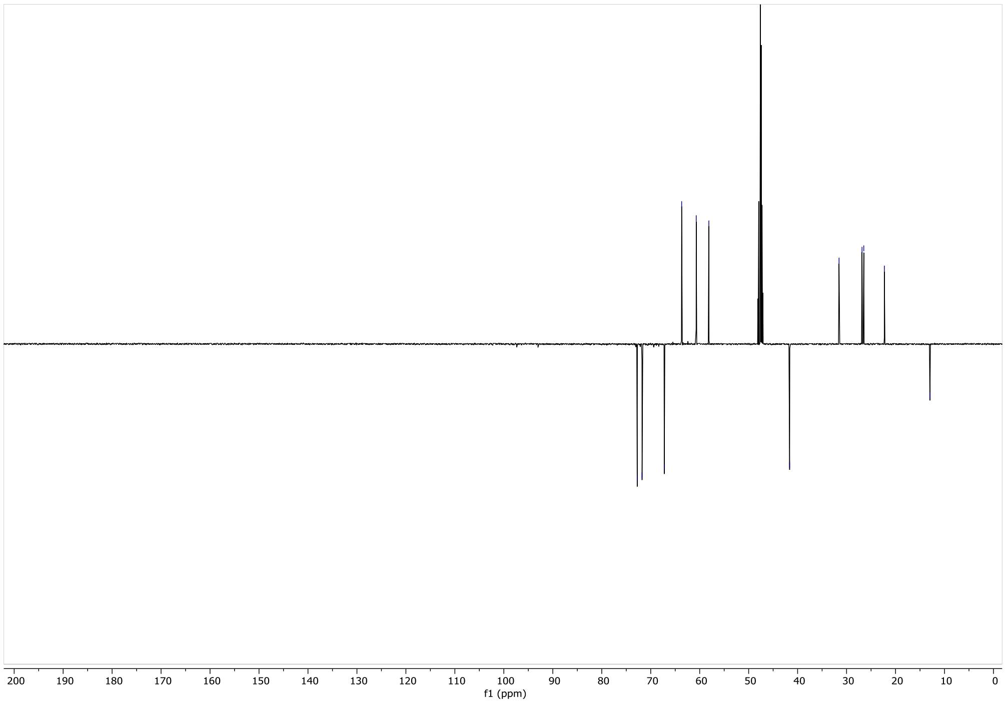

Figure S8: NMR data Ara8

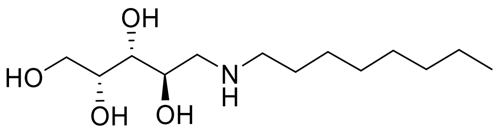

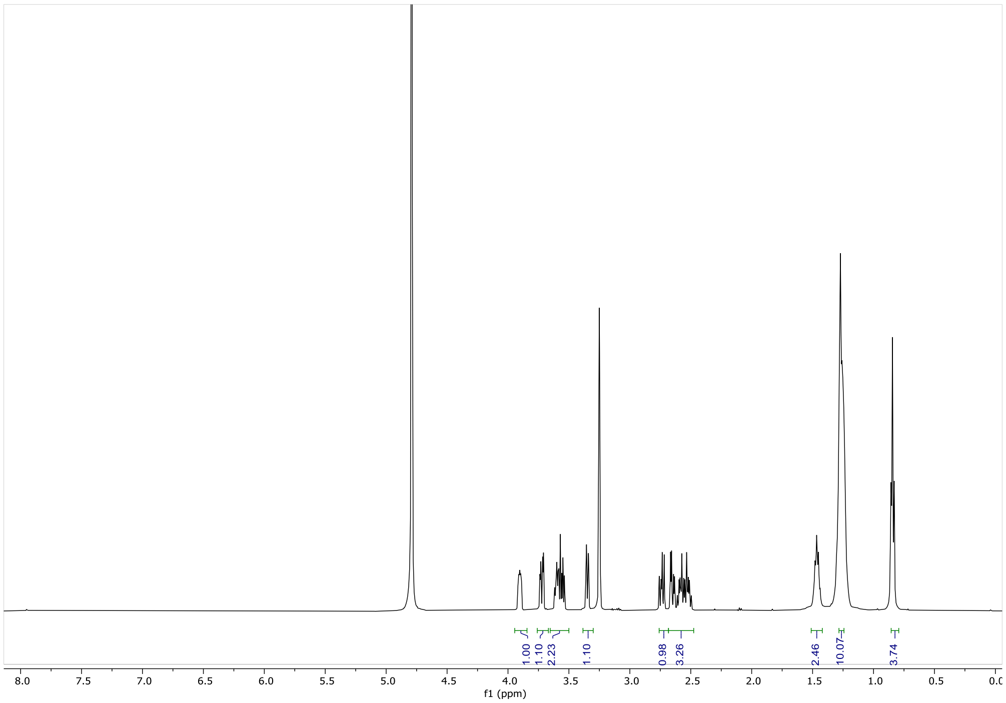

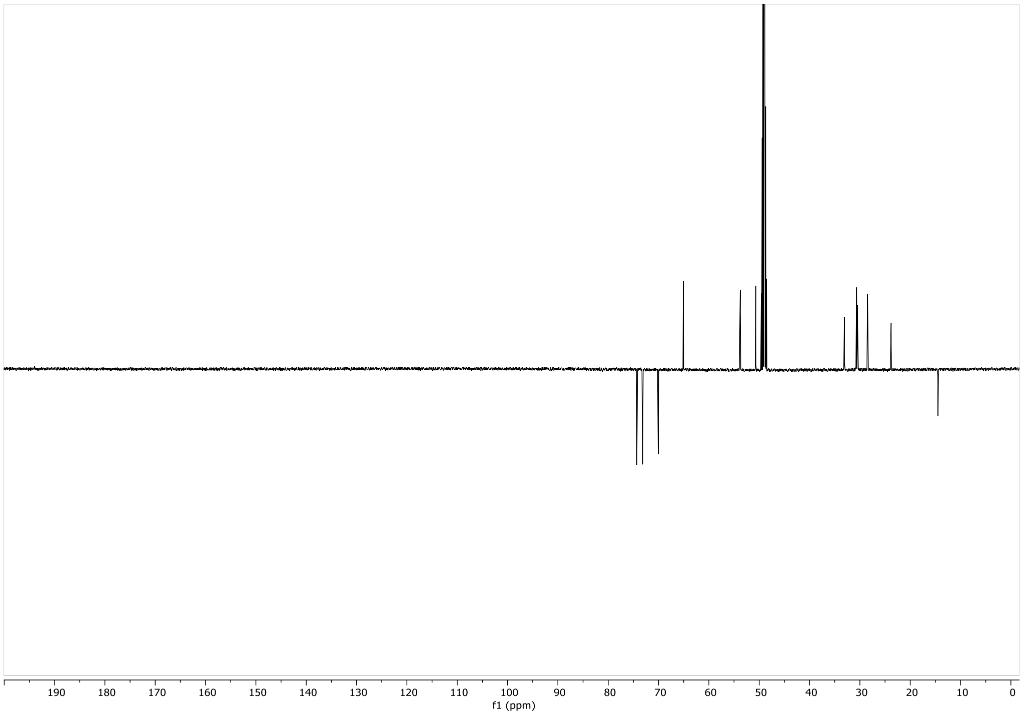

Figure S9: NMR data Ara1.8

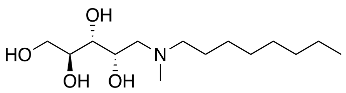

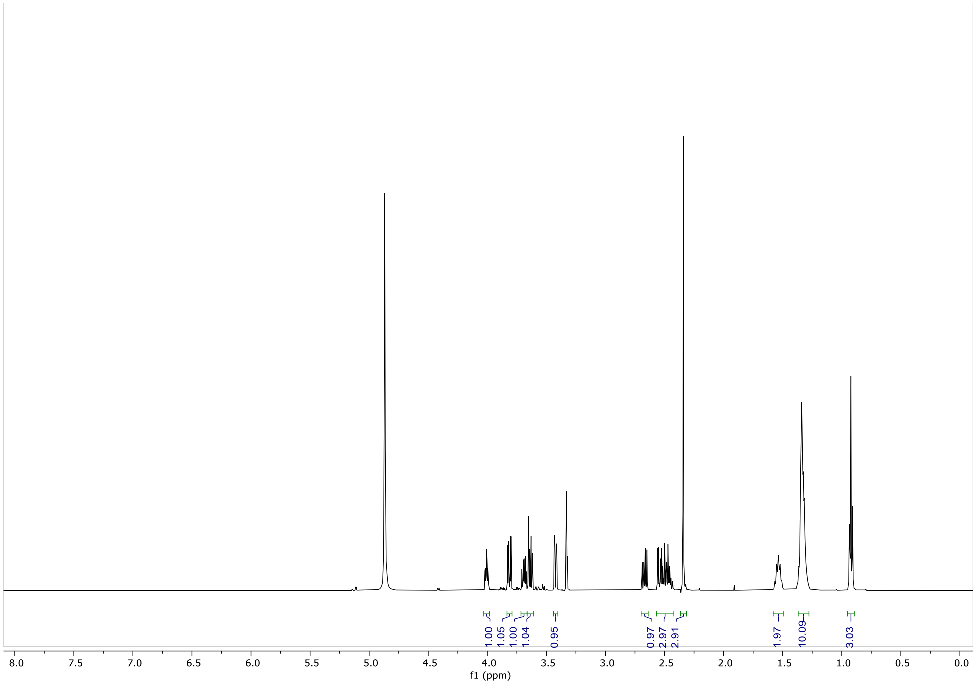

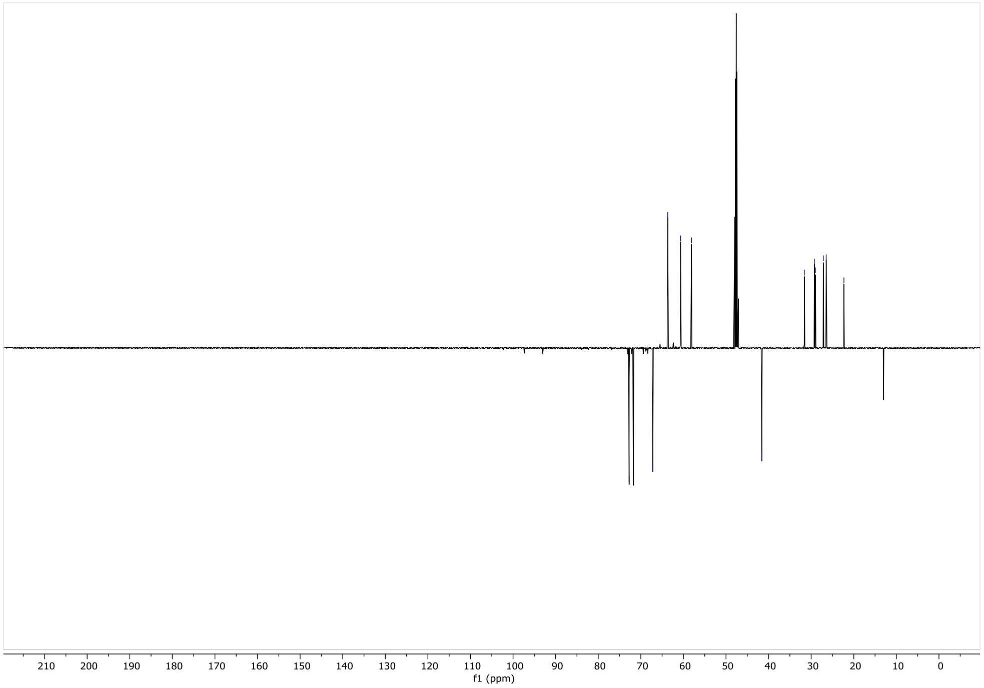

Figure S10: NMR data Ara10

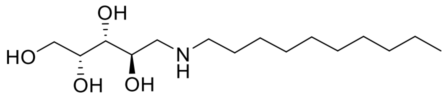

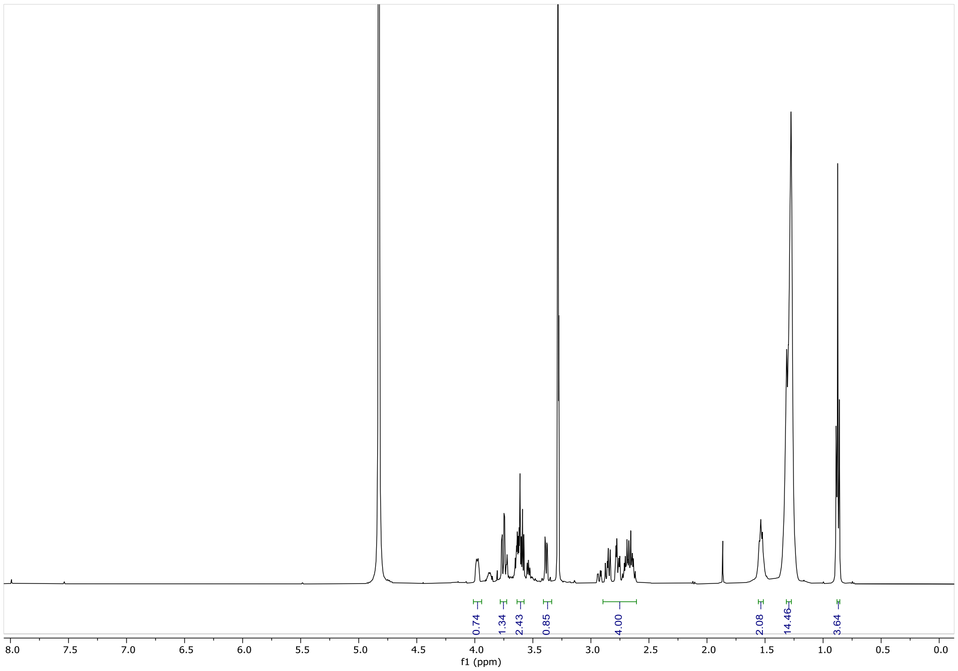

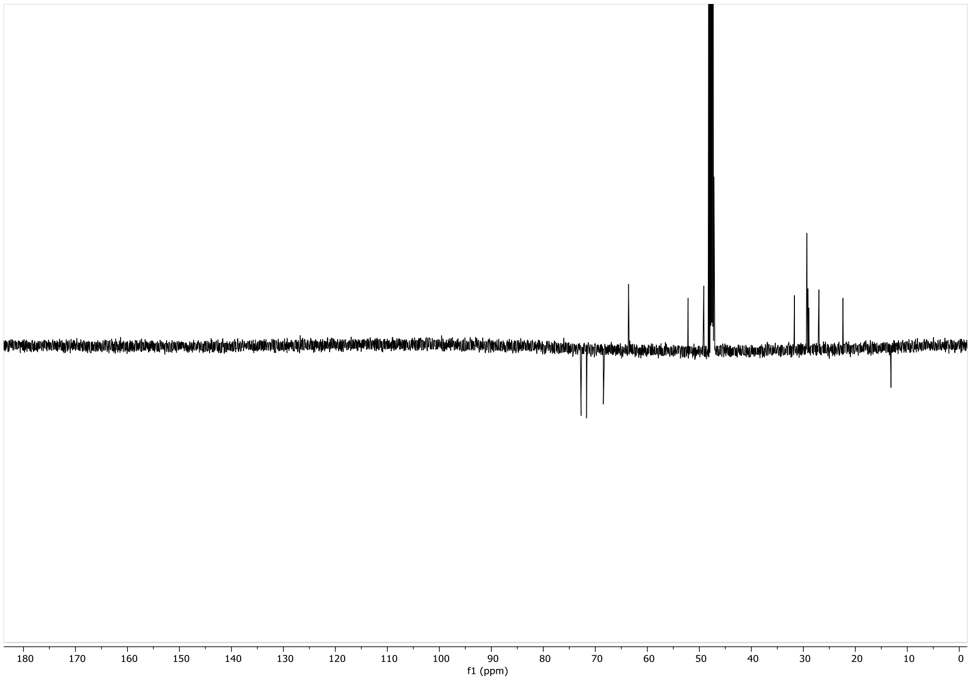

Figure S11: NMR data Ara1.10

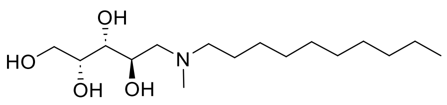

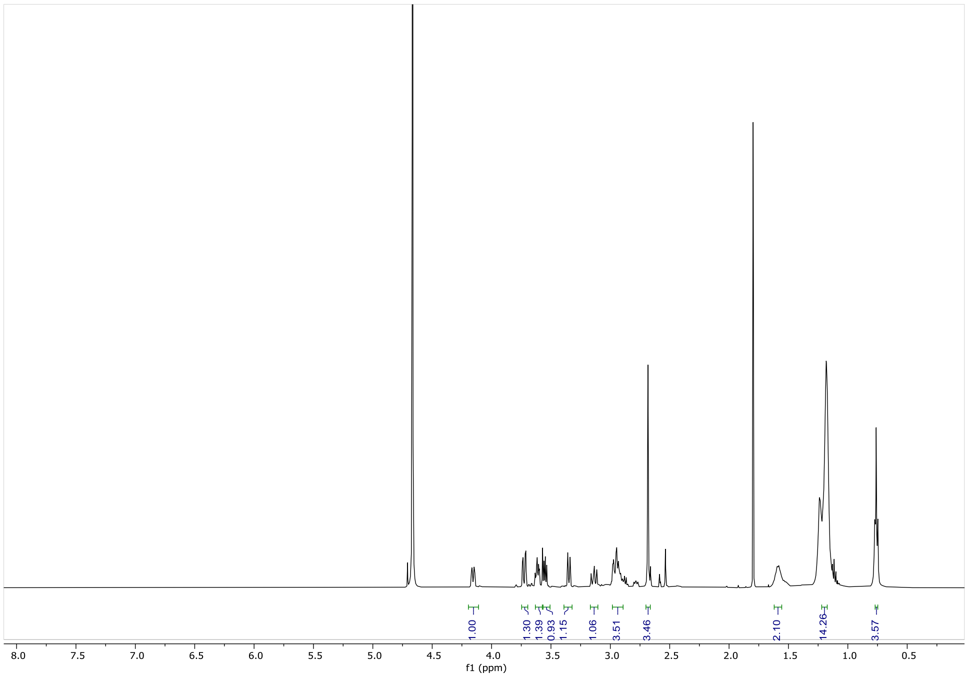

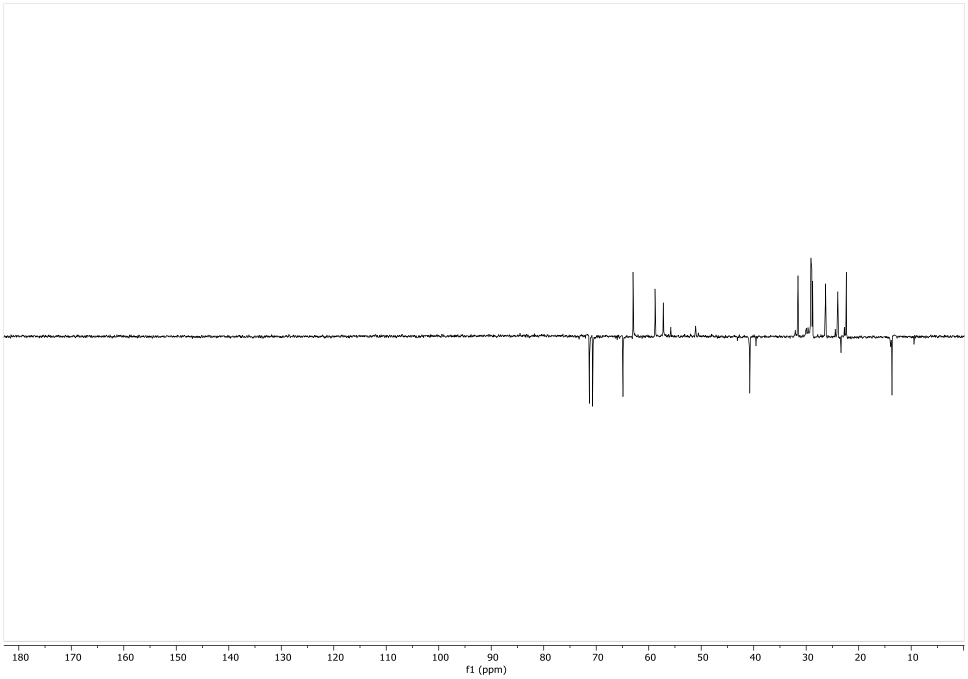

Figure S12: NMR data Ara12

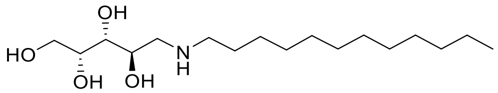

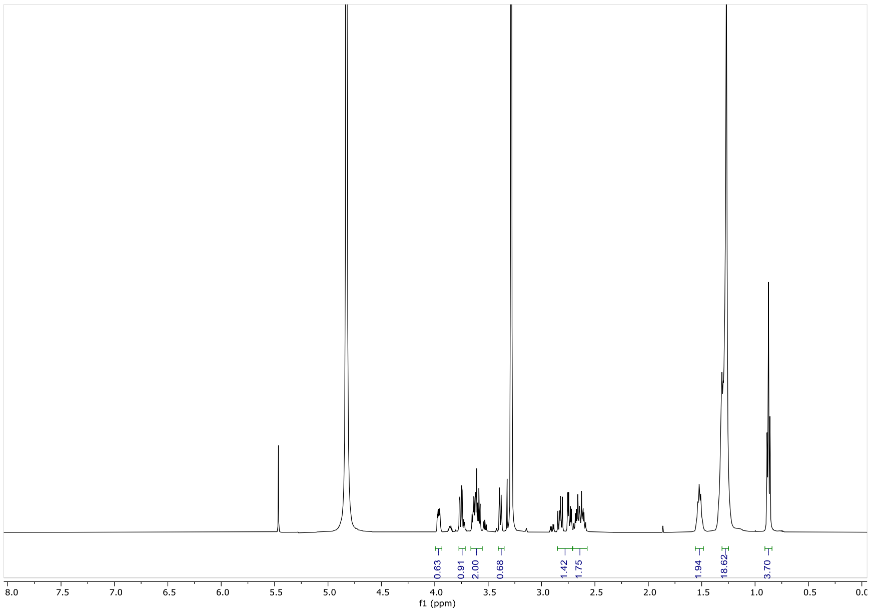

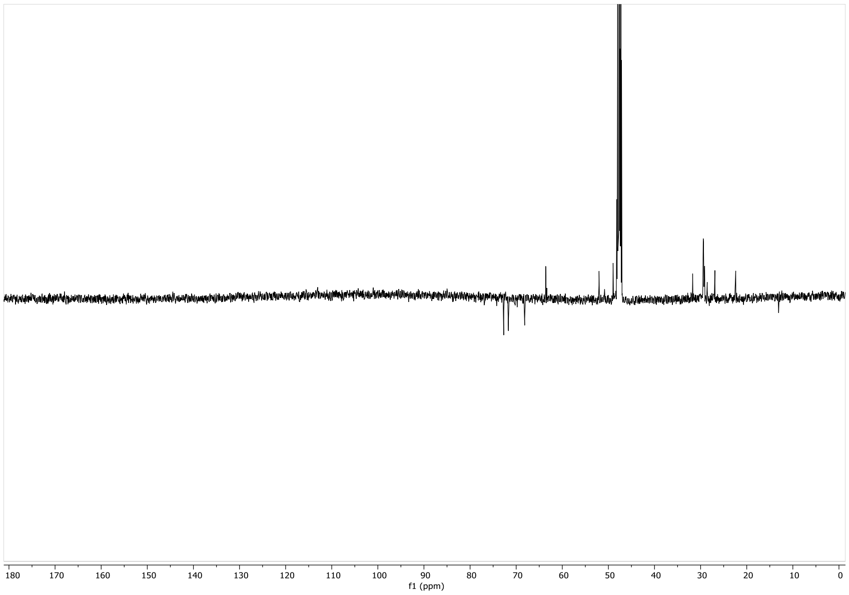

Figure S13: NMR data Ara1.12

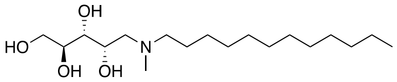

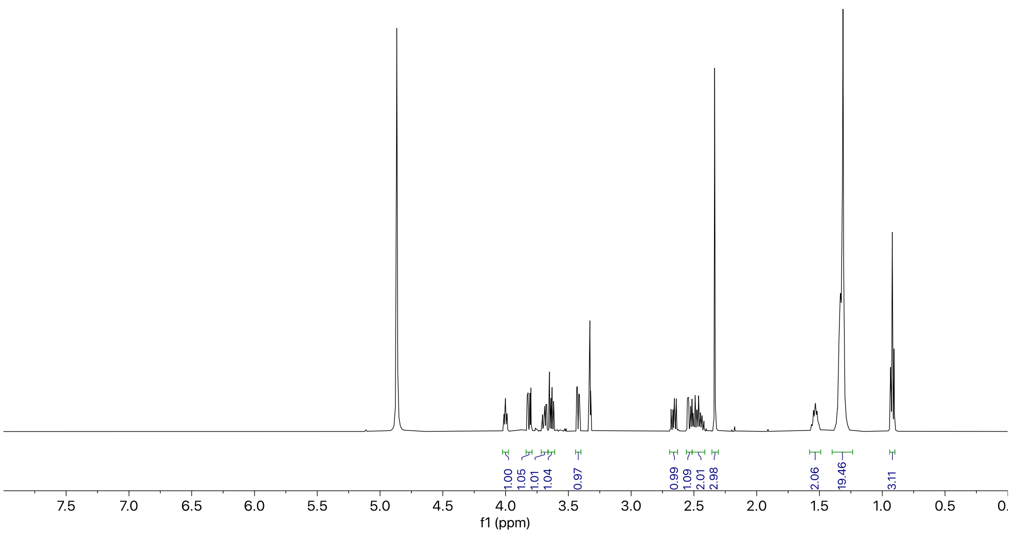

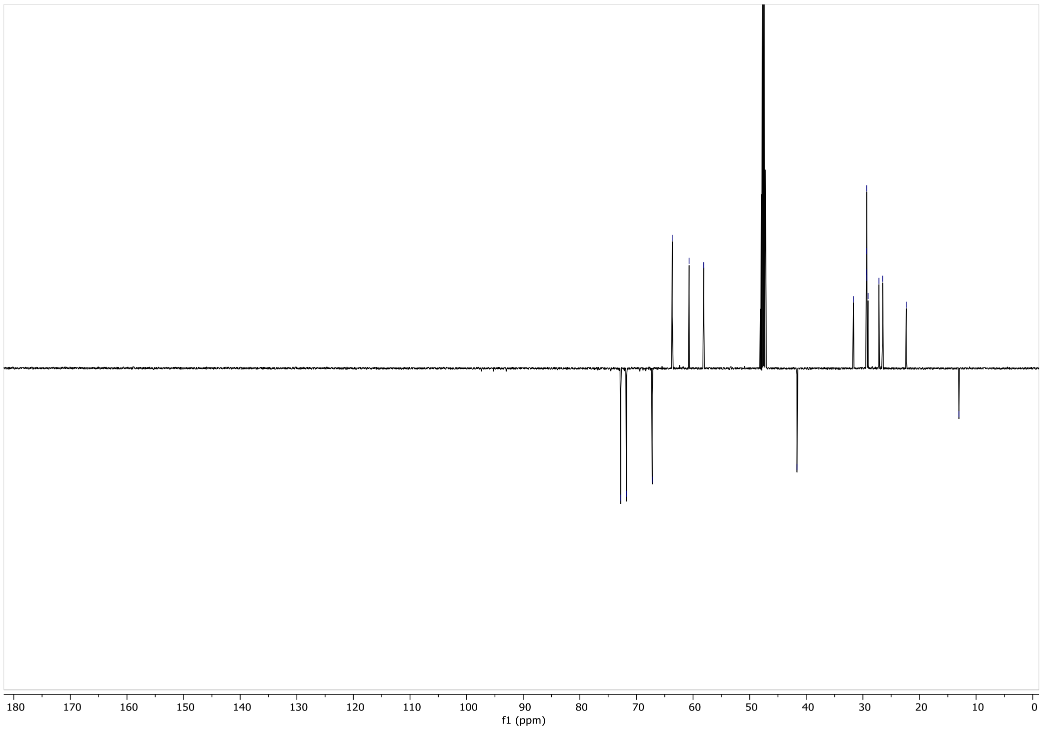

Figure S14: NMR data GalA6

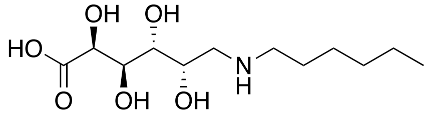

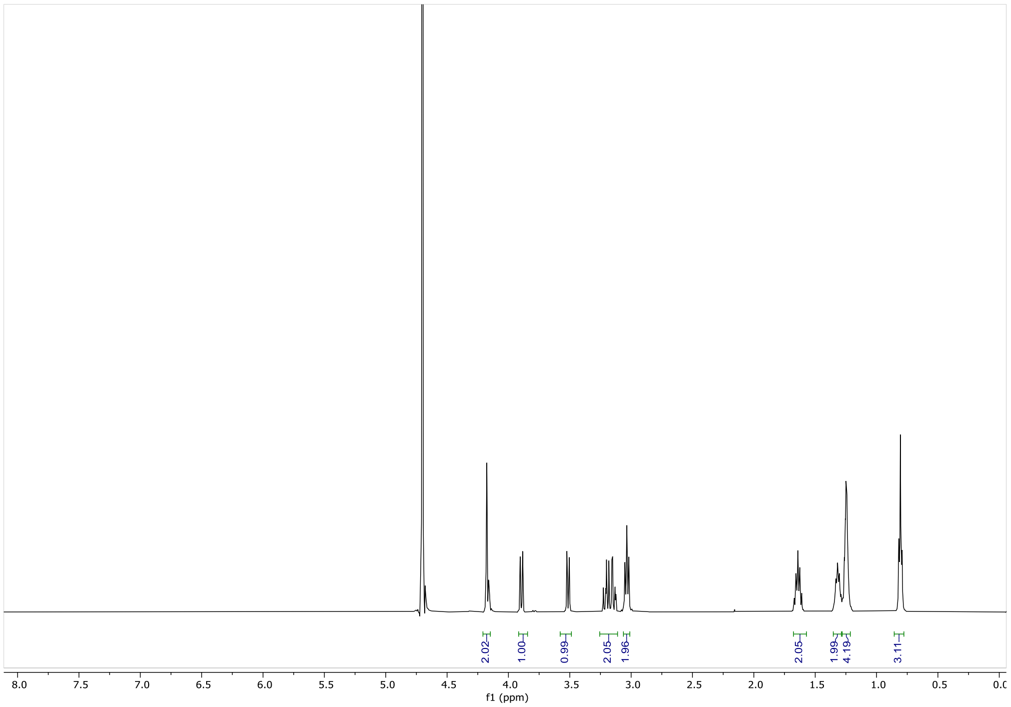

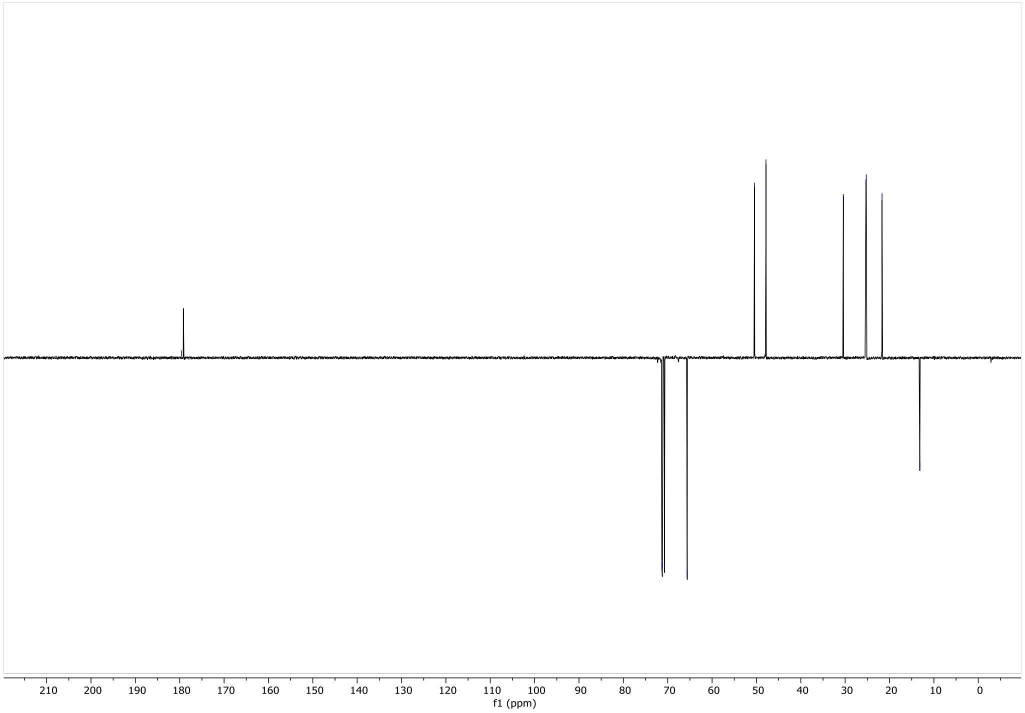

Figure S15: NMR data GalA1.6

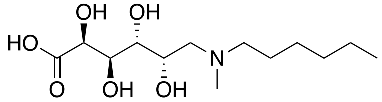

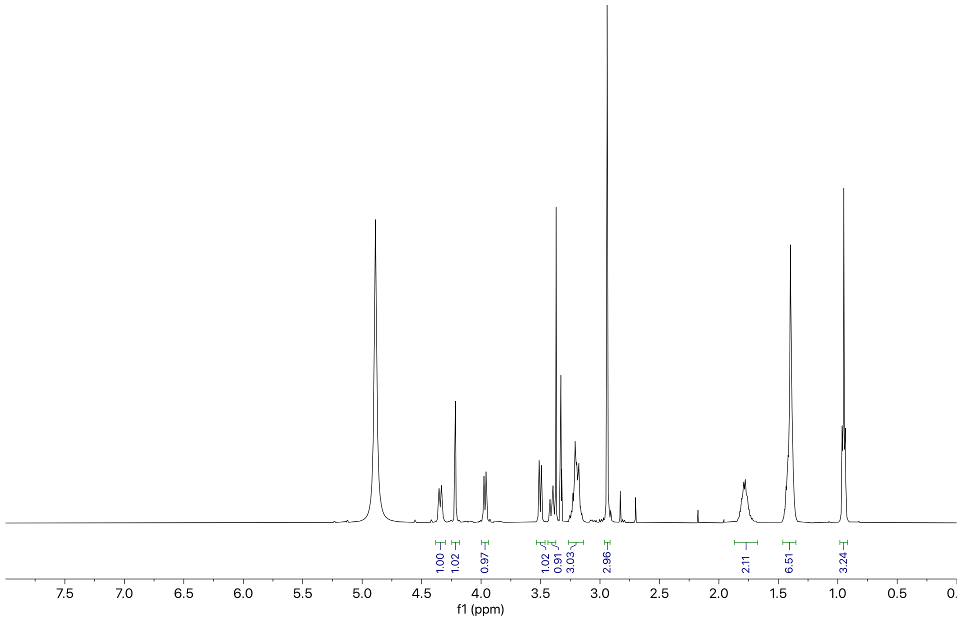

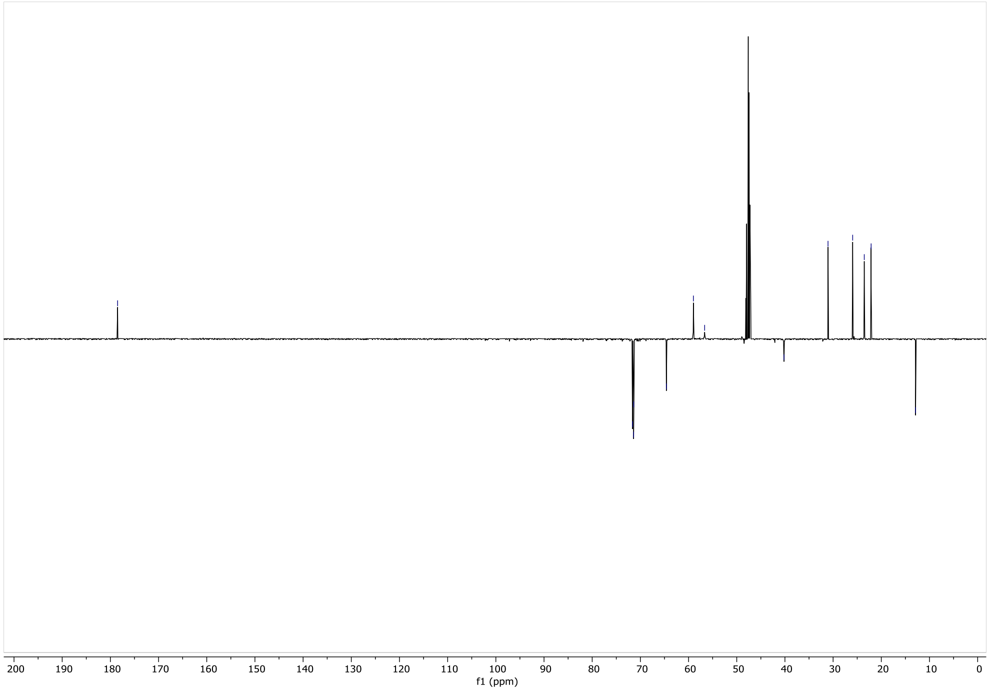

Figure S16: NMR data GalA8

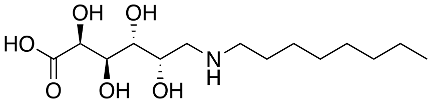

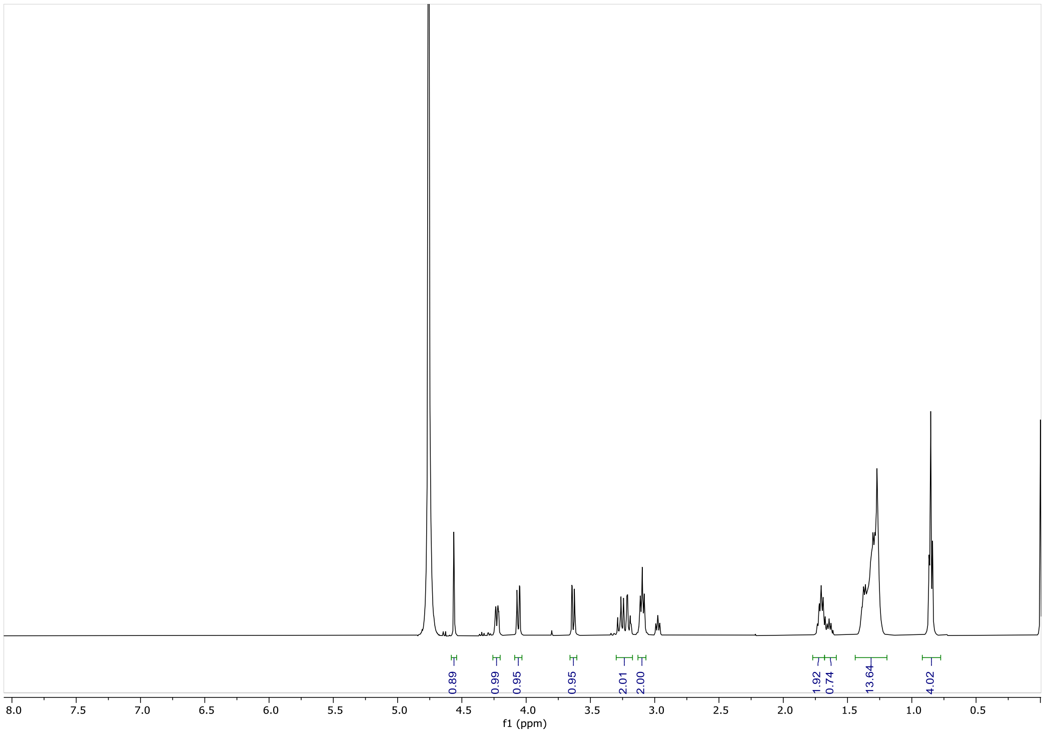

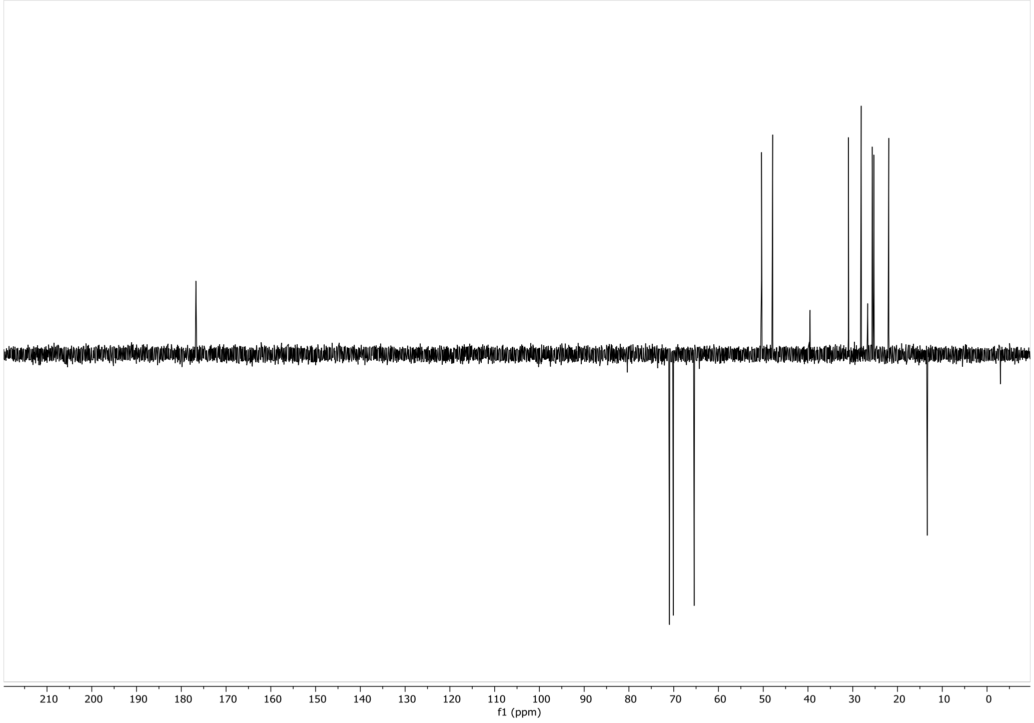

Figure S17: NMR data GalA1.8

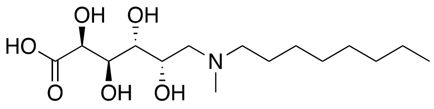

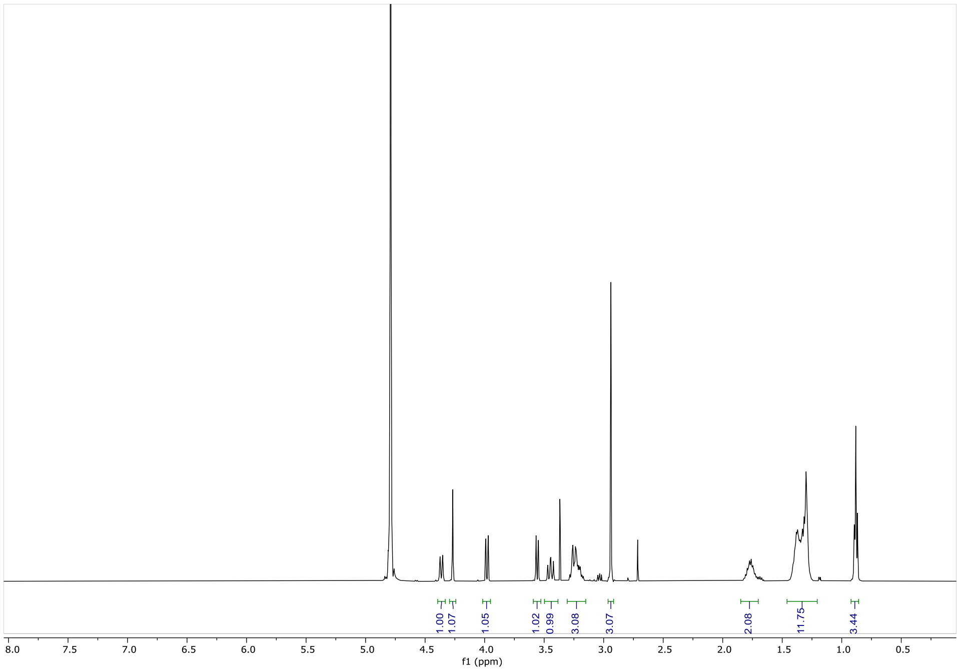

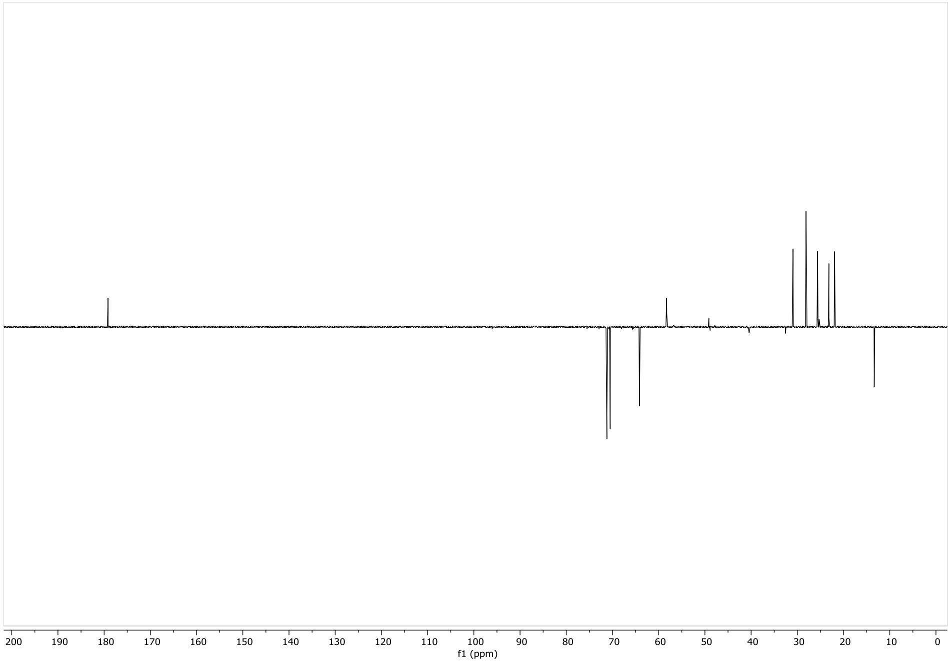

Figure S18: NMR data GalA10

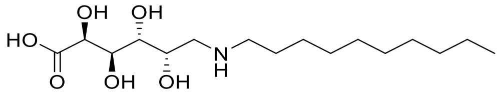

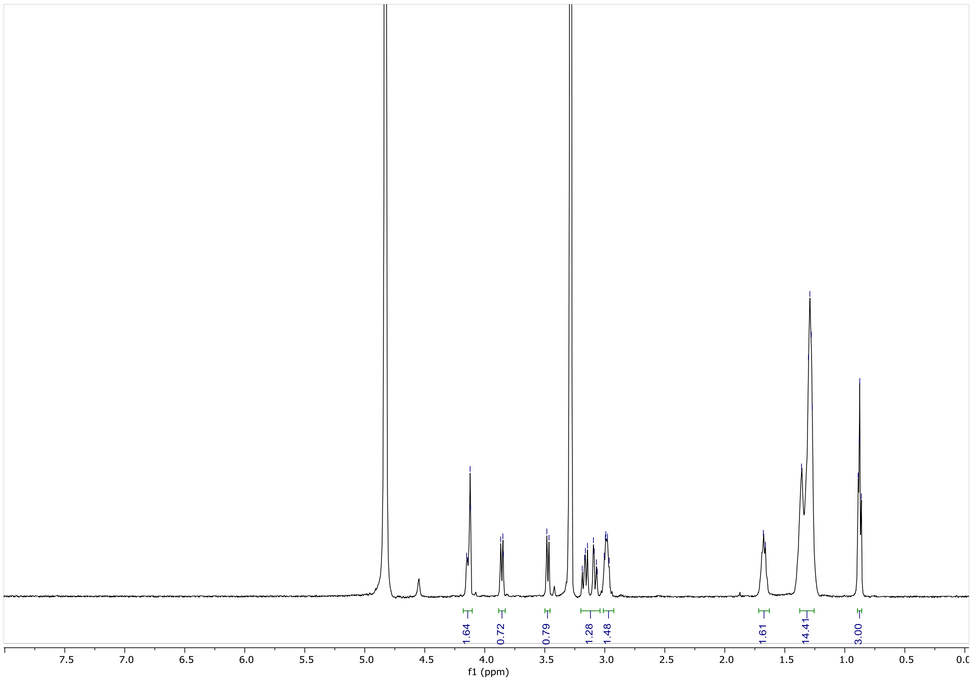

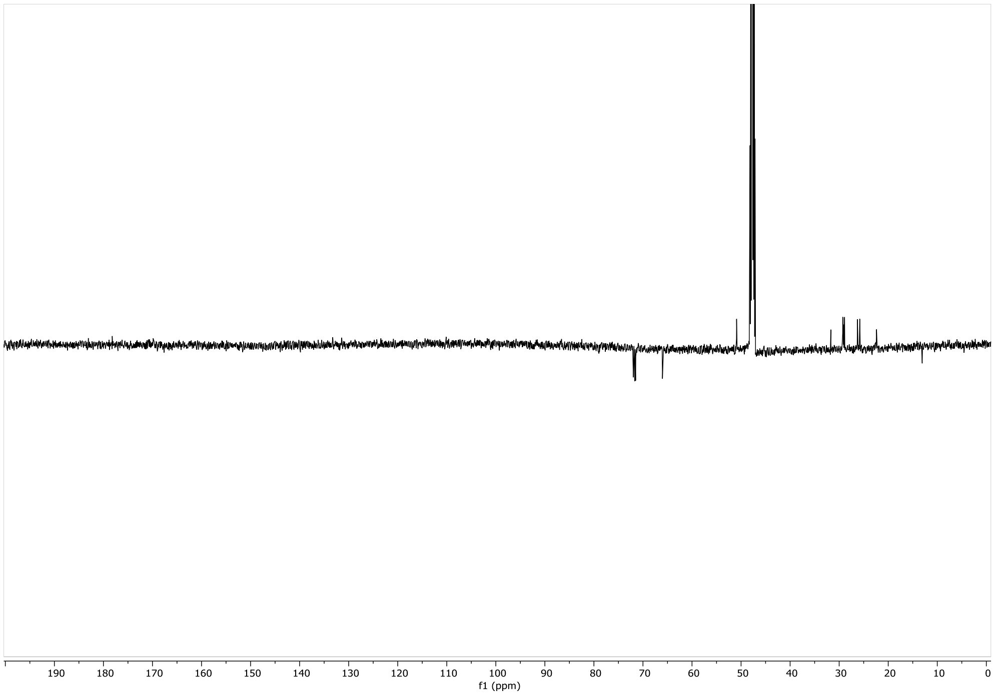

Figure S19: NMR data GalA1.10

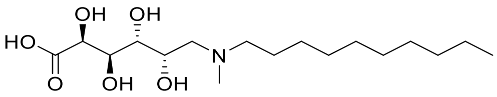

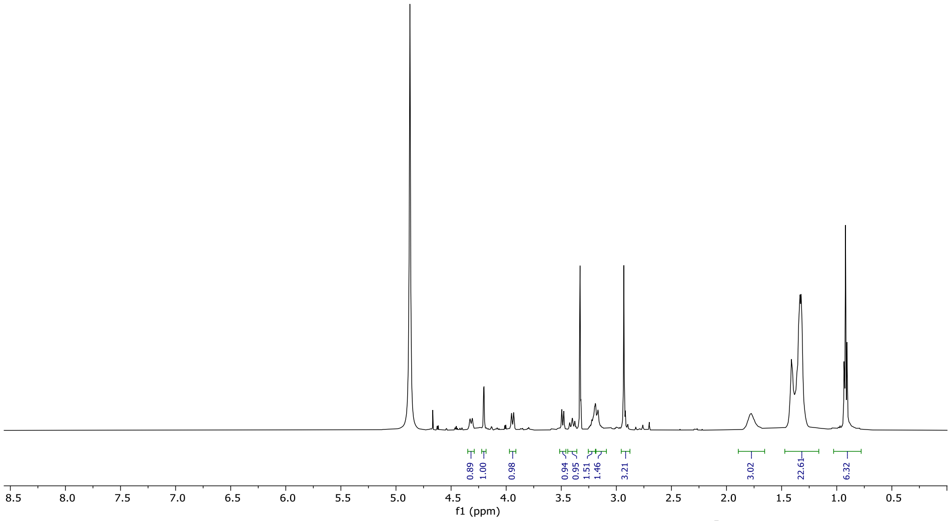

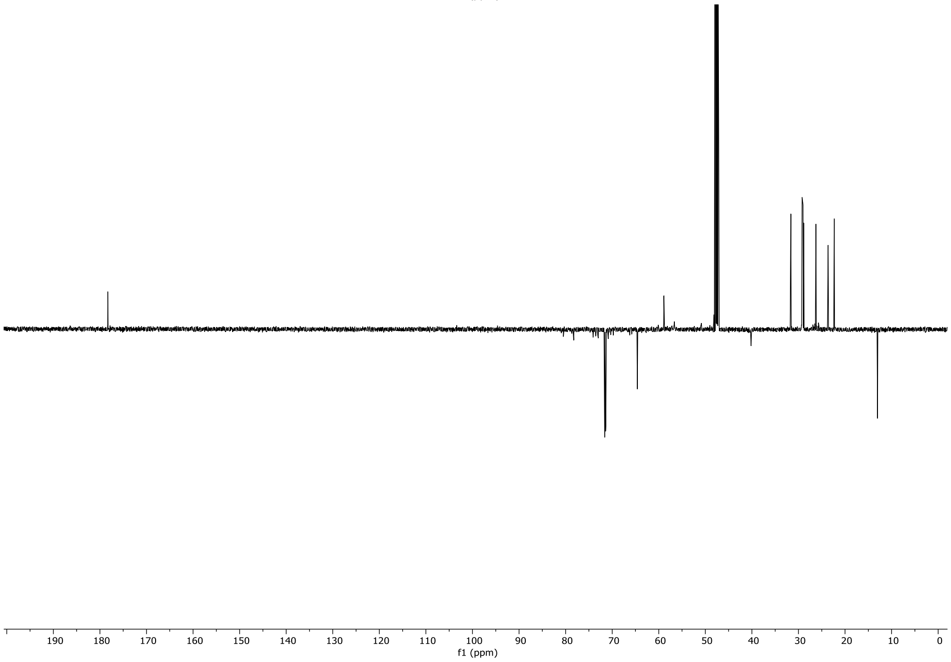

Figure S20: GalA12

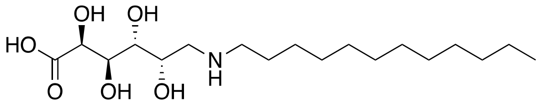

→ Does not dissolve, no NMR spectra

Figure S21: NMR data GalA1.12

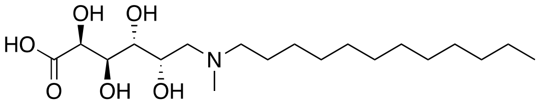

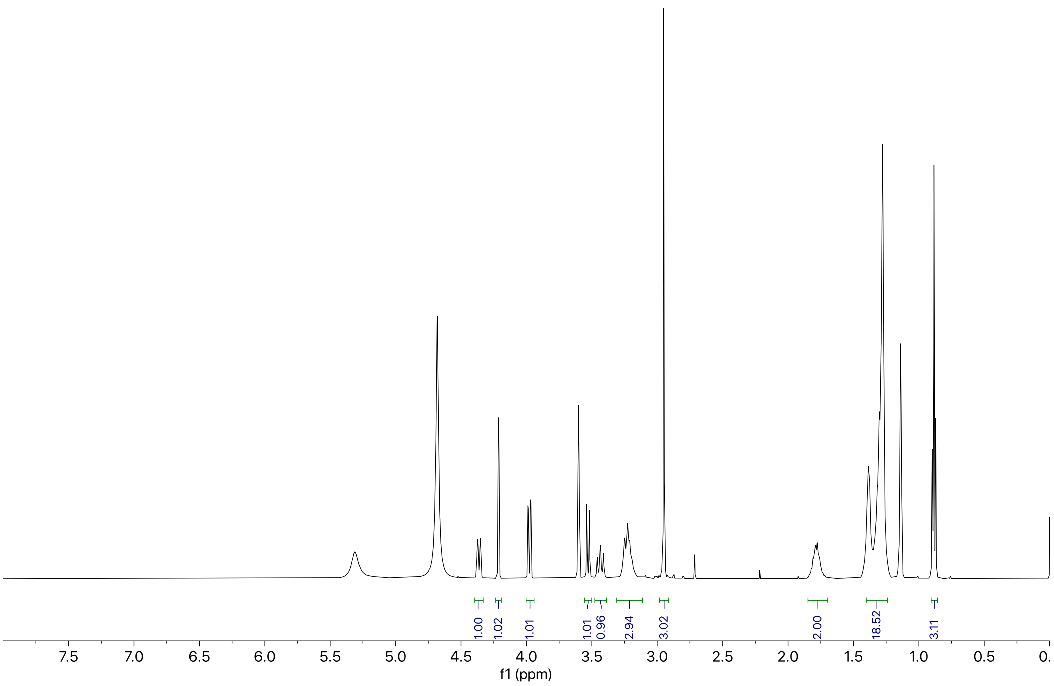

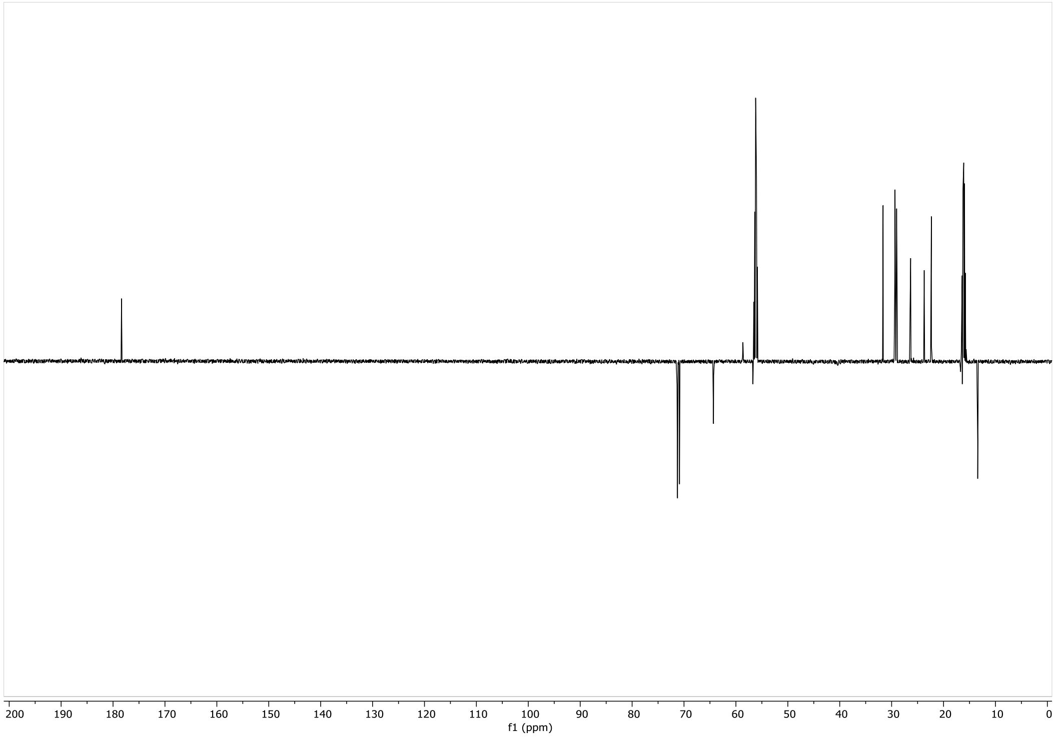

Supplement: Supplementary file 1 — sc3c03753_si_001.pdf [file sc3c03753_si_001.pdf]
